# The Encoding of Decision Difficulty and Movement Time in the Primate Premotor Cortex

**DOI:** 10.1371/journal.pcbi.1004502

**Published:** 2015-11-10

**Authors:** Marina Martinez-Garcia, Andrea Insabato, Mario Pannunzi, Jose L. Pardo-Vazquez, Carlos Acuña, Gustavo Deco

**Affiliations:** 1 Universitat Pompeu Fabra, Theoretical and Computational Neuroscience Center for Brain and Cognition, Barcelona, Spain; 2 Department of Ophthalmology and Institute of Neuropathology, RWTH Aachen University, Aachen, Germany; 3 Circuit Dynamics & Computation Laboratory, Champalimaud Neuroscience Programme, Lisboa, Portugal; 4 Departamento de Fisiología, Facultad de Medicina, Universidad de Santiago de Compostela, Santiago de Compostela, Spain; 5 Institució Catalana de Recerca i Estudis Avançats (ICREA), Barcelona, Spain; Istituto Italiano di Tecnologia, ITALY

## Abstract

Estimating the difficulty of a decision is a fundamental process to elaborate complex and adaptive behaviour. In this paper, we show that the movement time of behaving monkeys performing a decision-making task is correlated with decision difficulty and that the activity of a population of neurons in ventral Premotor cortex correlates with the movement time. Moreover, we found another population of neurons that encodes the discriminability of the stimulus, thereby supplying another source of information about the difficulty of the decision. The activity of neurons encoding the difficulty can be produced by very different computations. Therefore, we show that decision difficulty can be encoded through three different mechanisms: 1. Switch time coding, 2. rate coding and 3. binary coding. This rich representation reflects the basis of different functional aspects of difficulty in the making of a decision and the possible role of difficulty estimation in complex decision scenarios.

## Author Summary

Understanding how the brain produces complex cognitive functions has been a crucial question since ancient philosophical inquiries. The encoding of decision difficulty in the brain is fundamental for complex and adaptive behaviour, and can provide valuable information in uncertain environments where the future outcome of a choice must be evaluated beforehand. Here we show that neurons in premotor cortex represent the difficulty of a decision using at least three different variables: 1) the time of the neuronal response, 2) the intensity of the neuronal response, 3) the probability of switching from a low activity to a high activity profile. Moreover, we show that, by encoding the time elapsed from the end of the stimulus and commitment to a choice, another set of premotor neurons is able to provide information about the difficulty of the decision. These results show that the brain is implementing heterogeneous neural mechanisms to fulfill a complex cognitive function.

## Introduction

The information about the difficulty of a decision can be very valuable to properly allocate cognitive resources or to develop complex plans. Indeed, not only humans but even very simple form of life like honey bees are able to selectively avoid difficult decisions [1]. Moreover the degree of difficulty in a decision can also serve as a building block for the construction of confidence and to predict the outcome of a course of action.

Despite the relevance of the representation of difficulty very few is known about how the brain encodes and manipulate this information. It was shown that the onset and steepness of ramping activity in LIP depends on the amount of evidence for the decision [2], hence on the difficulty. This result was confirmed and extended by Ponce-Alvarez and colleagues [3], who found that in neural ensembles showing abrupt changes of activity both the time of the change and its variability depend on the difficulty of the decision. Pardo-Vázquez and colleagues [4] showed evidence of the effect of difficulty in PMv neurons, where a population of decision selective neurons is reported to have higher firing rate (FR) in easy compared to difficult trials for preferred correct choices and lower FR for non preferred correct choices. Also neurons in rats orbitofrontal cortex were found to modulate stimulus difficulty [5]. This article is indeed part of a growing body of literature [5–9] suggesting that single neurons in different brain areas (orbitofrontal cortex, lateral intraparietal sulcus, pulvinar) are involved in decision confidence processing. Although doubts can be cast on the metacognitive nature of the tasks employed [1, 10–14], these studies present evidence that the activity of neurons at least correlates with the difficulty of the decision.

However all these results still leave open the question as to how the neural signals of difficulty can be encoded in single trials. As a working hypothesis we expect that, during the decision process, the difficulty can be encoded in a continuous way by some feature of the decision process itself or by another monitoring process. Nonetheless we often take decisions based on the perceived difficulty of another decision (e.g. if it is too hard to tell if somebody is bluffing at poker a player could decide to leave the trick). Therefore we also expect that the difficulty signal can be used by another decision process and in this case a discrete representation may arise. According to this general hypothesis both continuous and discrete representations could be implemented in the brain. In consequence, it remains unclear what kind of encoding is used in the brain to represent difficulty.

Therefore in the present study our aim is to shed light on the possible mechanisms used by the primate brain to represent decision difficulty. We have looked at how difficulty can be represented in single neurons recorded from ventral premotor cortex (PMv) while monkeys perform a visual discrimination task [4].

The correlation of reaction time (RT) and difficulty has been shown by many experimental [15–17] and explained by theoretical studies [18, 19]. In our experiment, given the experimental protocol, we can only record the movement time (MT) and not the RT. We hypothesize that MTs are related to the difficulty and tested this hypothesis. Indeed it was already shown [4] that MT is different in easy compared to difficult choices. Here we found that the MT correlates with the difficulty of the decision task. Therefore neurons encoding the MT could also bring information about the difficulty and the decision process itself. Indeed we found a neural correlate of MT in PMv. Moreover we report another population of neurons whose activity correlates with the discriminability of the stimulus and we investigate the computational schemes underlying this correlation. Our results suggest that both continuous and discrete coding schemes could be active in the brain.

## Results

### Neural correlate of movement time in PMv

**Fig 1:**
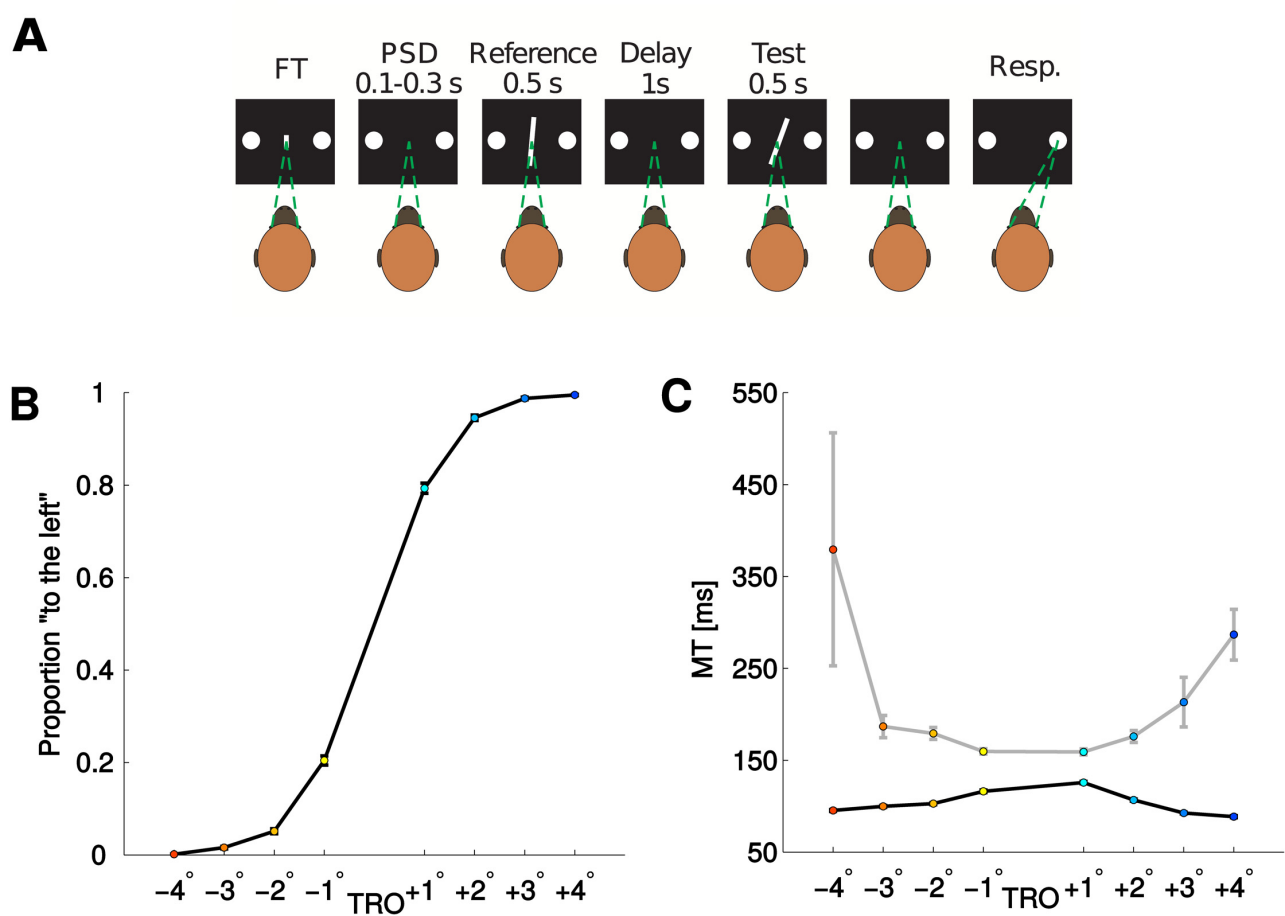
Panel A: Experimental paradigm. The trial starts when the monkey fixes the gaze to the central target (FT). A brief pre-stimulus delay follows (PSD). The reference bar is presented for 500 ms with one of three possible orientations (Reference). During the subsequent delay the subject has to maintain fixation (delay). The test bar is shown tilted to the left or to the right in relation to the reference bar. The amount of orientation of the test bar relative to the reference (TRO) manipulates the difficulty of the trial. When the test bar disappears the subject decides whether the test was tilted right or left in relation to the reference bar by making a saccade towards the right or left choice targets respectively. For more details see [4]. Panel B: Average probability to select the “left” option for the two monkeys as a function of the TRO. To each value of TRO a different color is assigned: Colors from dark blue (red) to light blue (yellow) correspond to left (right) responses with increasing difficulty, i.e., relative orientation from 4° (−4°) to 1° (−1°). Panel C: Average Movement time (MT) for the two monkeys as a function of the TRO separated for correct (black) and error (gray) trials.

We studied the decision-process in the primate brain during a simple binary decision task. Two male monkeys (Macaca mulatta) performed a two-interval two-alternative discrimination task. They were trained to compare the orientation of a reference bar (with variable orientation), presented during the first interval, with that of a test bar, presented during the second interval. Their task was to decide whether the test bar was tilted right or left as compared to the reference bar (see Fig 1A and Methods for details). The level of difficulty of the task was controlled by varying the difference between the orientation of the first and the second bar, i.e. the test bar’s relative orientation (TRO). The TRO was varied from one up to four degrees and in both directions. Consequently the choice of the subjects was affected and achieved almost perfect performance for TRO = 4° and TRO = −4°, as shown in Fig 1B.

Single cells from PMv were recorded while monkeys performed the task. For a more detailed description of the task, behavioral results and neural recordings see Methods and [4].

Our first objective was to find neural signatures of difficulty computations in PMv of the primate brain. It is plausible that these computations take place in the same area as where the decision is encoded. In addition, it has been shown evidence of different neural dynamics in a decision-related PMv population in easy compared to difficult choices [4]. We therefore analyzed the activity of PMv activity recorded during the decision task. Our analysis was restricted to a subset of the recorded neurons (324 neurons, see [4]), comprising the cells that were relevant to the decision task.

The correlation between difficulty and RT has been well established, both by experimental [15–17] and theoretical [18, 19] works. In our experiment we can only record the movement time (MT) and not the RT. We hypothesize that MTs are related to the difficulty and tested this hypothesis. The MT is the time from the end of the second interval (subjects were not allowed to chose one option before the end of the second interval) until the response of the subject. The MT must not be confused with the decision time, indeed the decision could be taken before the end of second interval (even though MT and decision time could be correlated). However, there is a big variability in MTs and they could therefore be informative about the difficulty of the decision. Here we want to test the hypothesis that MT are correlated with the difficulty of the decision.

As shown in Fig 1C, MTs decrease as a function of the ease of the trial in correct trials and increase in error trials (Pearson correlation coefficients: TRO< 0, correct: 0.11; TRO> 0, correct: -0.18; TRO< 0, error: -0.13; TRO> 0, error: 0.14; all p-values< 10^−7^). This X-shaped pattern has been previously associated to decision confidence [5, 7, 20–22]. However we note that this pattern is neither necessary [14, 23, 24] nor sufficient to define confidence, since it could also emerge from different computations. Therefore and since we don’t have a direct measure of confidence in our task we will state that the MT, like the RT or decision time, is informative about the difficulty, leaving open the question whether it could represent a signature of metacognitive processing.

**Fig 2:**
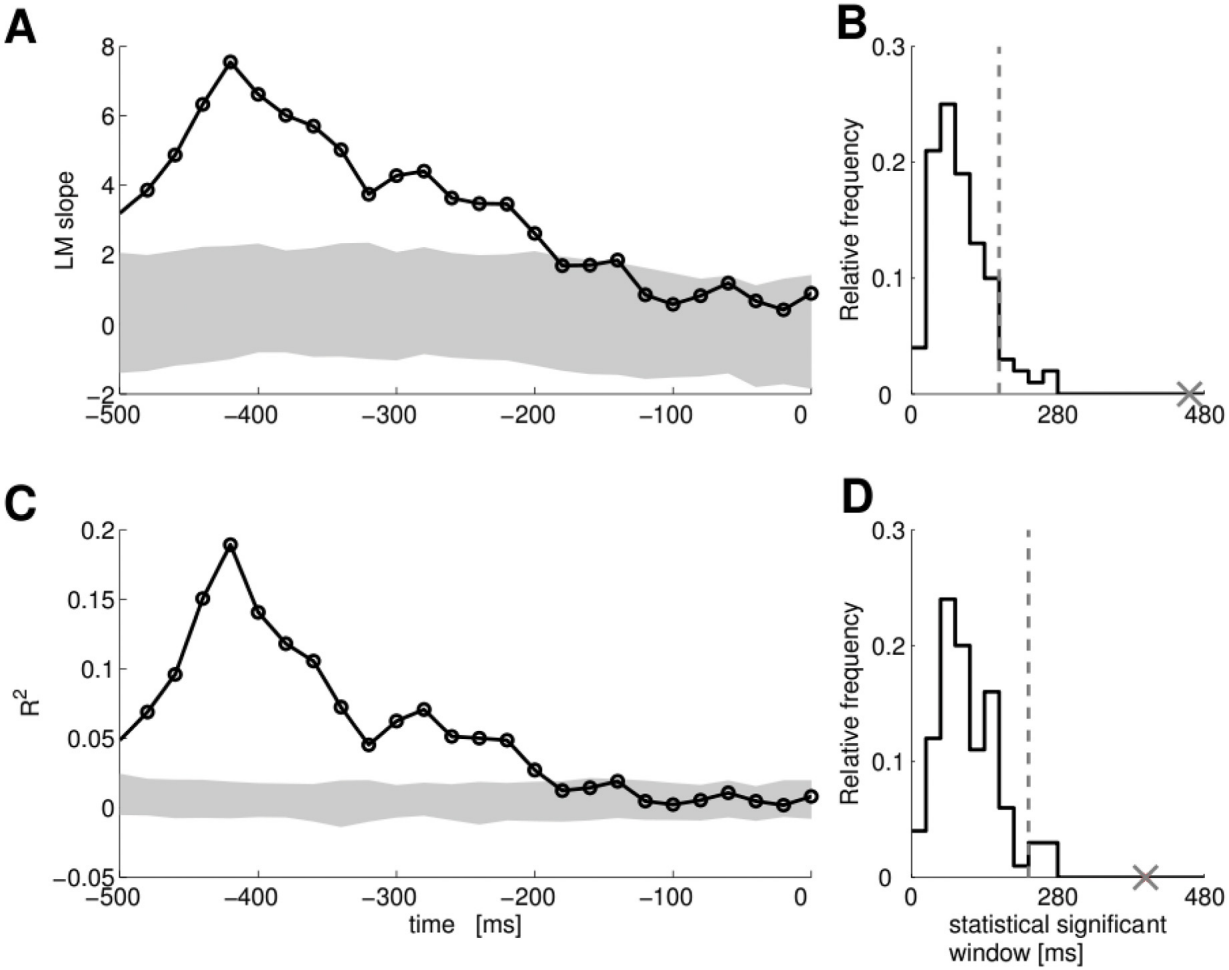
PMv single neuron whose activity correlates with MT. Panel A: The time evolution of the *LM*_*MT*_ slope coefficient. Shaded area represents the 95% of the distribution of the slope under the null hypothesis (Monte Carlo random resampling, *p* < 0.05). Trials for this panel and panel D are aligned to the saccade; around −510 ms the second bar was shown. Panel B: The distribution under the null hypothesis of the number of consecutive bins. The cross indicates the number of consecutive bins for the single neuron data in panel A, and the vertical dashed line indicates the 95% threshold. The x-axis is shown in ms for an easy comparison with panel A. Panel C: The time evolution of the R^2^ for the same single neuron. Shaded area represents the 95% of the distribution of the R^2^ under the null hypothesis (Monte Carlo random resampling, *p* < 0.05). Panel D: The distribution under the null hypothesis of the number of consecutive bins. The cross indicates the number of consecutive R^2^ bins for the same single neuron, and the vertical dashed line indicates the 95% threshold.

We used a linear regression model (*LM*_*mt*_, see Methods) to test whether neurons in PMv encode the MT. In Fig 2 the linear analysis of a PMv single neuron is shown whose FRs encodes MT. Line in Fig 2A shows the slope of the regression line for a single neuron.

The significance of the slope of the regression line was assessed by Monte-Carlo random resampling (*p* < 0.05). The shaded area in Fig 2A represents the 95% of the distribution of the slope under the null hypothesis. Values of the slope falling in the range indicated by the shaded area considered as not significant since they are indistinguishable from values obtained by chance. Moreover a minimum number of consecutive significant bins is required in order to avoid false positives due to fluctuations. The minimum number of consecutive significant bins was chosen based on the distribution of consecutive significant bins under the null hypothesis. This probability distribution was estimated by Monte-Carlo random resampling. Fig 2B shows the distribution under the null hypothesis for the same single neuron of panel A (shown in ms for an easy comparison with panel A). We considered a neuron to have a significant slope of the regression line only if its longest interval of consecutive bins had a low probability under the null hypothesis. We believe that this strict requirement is necessary for this type of data, since the time correlation of FRs can affect the significance of results. Since we applied this test to the whole population we controlled the false discovery rate (FDR) with Benjamini-Hochberg procedure [25]. We show in S1 Fig the results of controlling FDR at varying values of *Q*. We observe that in a wide range of *Q* a large amount of true discoveries is made. For *Q* = 0.05, for example, we found 276 neurons (82% of the analysed population), such that the slope of the *LM*_*mt*_ is significantly different from that given by chance. We note that this is a lower estimate of the number of neurons encoding MT in the dataset (as explained in S1 Text). We can choose such a small value in order to limit the number of false discoveries, however such a low value for *Q* can induce a large underestimate of the number of false null-hypotheses (as shown in S1 Text).

Even if we found many neurons with a significant slope, it is still possible that the *LM*_*mt*_ is explaining a small portion of the variance of the data from those neurons. In order to rule out this possibility we analysed the coefficient of determination (R^2^) of the *LM*_*mt*_. Fig 2D shows the R^2^ and the 95% of its distribution under the null hypothesis for the same single neuron of panels B and C. We applied to *R* ^2^ the same procedure used for the slope of the regression line (see Methods) and took a minimum number of consecutive significant bins (the distribution of consecutive significant bins under the null-hypothesis is shown in Fig 2D). We found that, for 248 neurons out of 276, the value of the *R* ^2^ is significant, even though the values of the R^2^ for the 248 neurons are quite small (99% of the distribution is between 0 and 0.23). To our knowledge this method has not been used until now in the analysis of neurophysiological recordings and could set a new standard for analysing single neurons (see Methods for details). In summary we found 248 neurons in PMv, whose activity is informative about the MT.

### Representation of the difficulty of decision in PMv neurons

We want to test here the idea that PMv neurons directly represent the discriminability of the stimulus and hence the difficulty of the decision. In the following single neurons analyses we used only correct trials, since error trials were not enough to produce clear results. We found a population of neurons whose FR was informative about the difficulty of the task for at least one of the two possible choices (as revealed by the linear regression model *LM*_*tro*_; same method as for the MT analysis above was applied, see Methods). Each of these neurons was able to encode the difficulty of the task only for one choice when isolated but their population signal can be integrated by downstream neurons.

**Fig 3:**
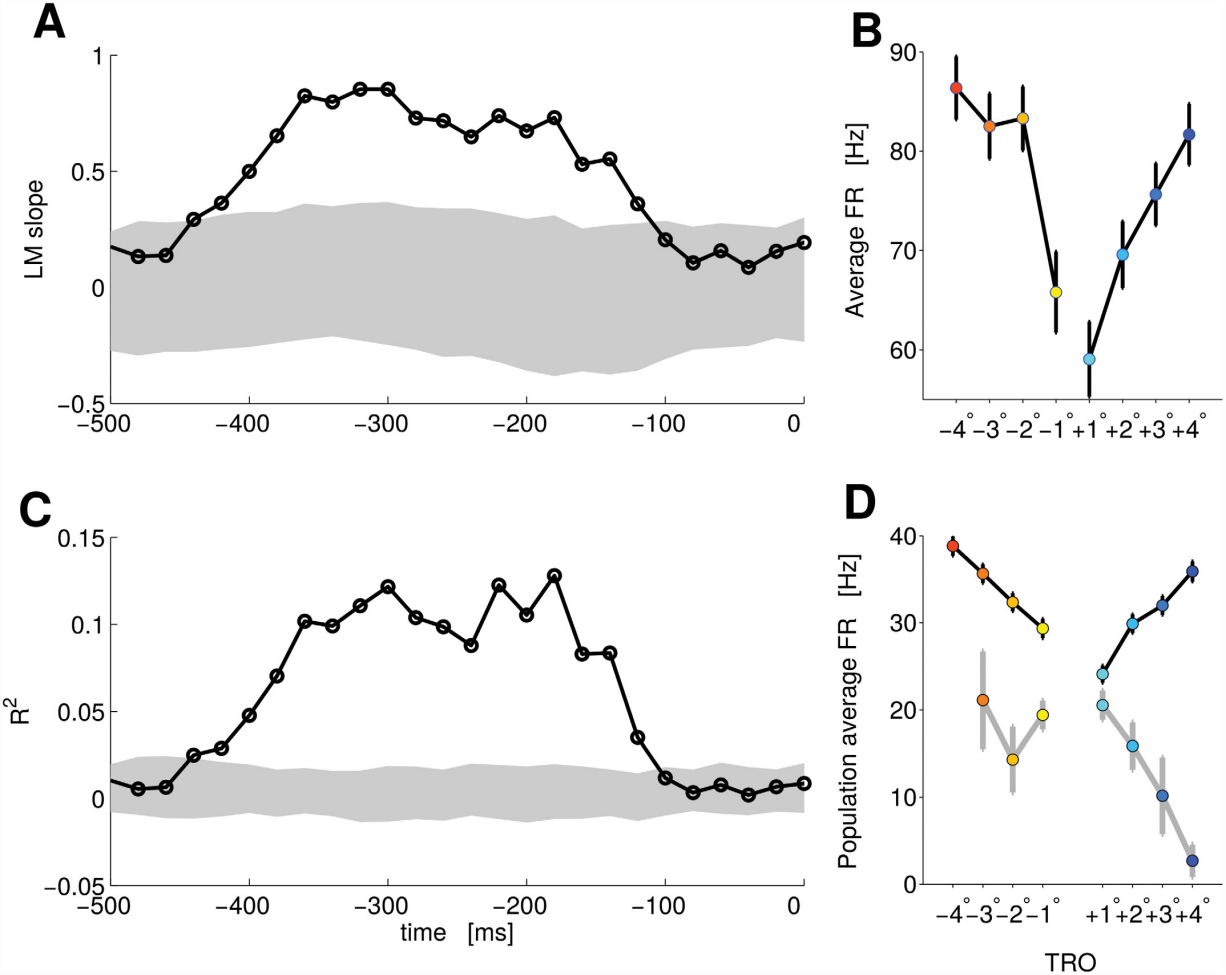
PMv single neuron and population activity correlating with the discriminability. Panel A: The time evolution of the *LM*_*diff*_ slope coefficient. Shaded area represents the 95% of the distribution of the slope under the null hypothesis (Monte Carlo random resampling, *p* < 0.05). Trials for this panel and panel D are aligned to the saccade; around −510 ms the second bar was shown. Panel B: Average FR as a function of TRO for the same neuron of panel A. The time average is taken during the time where the slope and the R^2^ are significant. Panel C: The time evolution of the R^2^ for the same single neuron. Shaded area represents the 95% of the distribution of the R^2^ under the null hypothesis (Monte Carlo random resampling, *p* < 0.05). Panel D: Average FR for the whole population (66 neurons) encoding difficulty (see text). Correct (black line) and error (gray line) trials are presented separately. The color code for the TRO is the same as Fig 1. Error bars of panels B and D represent SEM.

Hence we identified among these difficulty neurons, a population whose activity was similar for right and left decisions. The FR of these neurons encoded the difficulty of the task, independently of the subject’s choice, as revealed by the linear model (*LM*_*diff*_) (see Methods for details). Fig 3A shows the evolution in time of the slope of the regression line for a single neuron of this population. The shaded area represents the 95% of the distribution of the slope under the null-hypothesis (Monte-Carlo random resampling). The values of the slope of the regression line overlapping with the distribution of randomly resampled data are not significant, since they are indistinguishable from values given by chance. Values outside shaded area are considered significant. As shown in Fig 3B, in the time window where the slope is significant the FR of the neuron increases for both positive and negative values of TRO, producing a V-shaped pattern. Using this method we found 107 neurons for which the slope of the regression line was significant (*Q* = 0.1, Benjamini-Hochberg procedure, see Methods and S1 Text).

Fig 3C shows the R^2^ values and the 95% of its distribution under the null-hypothesis (Monte-Carlo random resampling). This result further constrains the encoding time window to regions where the R^2^ is significant. When we applied the R^2^ analysis only 66 neurons revealed to carry significantly more information than chance (*Q* = 0.1). This strong reduction in the number of informative neurons suggests that simple tests based on the slope of the regression line can be made more robust by applying also tests based on R^2^ to filter out elements that bring few linear information.

In summary we found 101 neurons encoding the difficulty of the task, or the TRO, for at least one of the two behavioural responses and 66 neurons encoding the difficulty for both behavioural responses (*Q* = 0.1).

In Fig 3D we show the population activity of these 66 neurons. When the behavioral response was incorrect (gray line) the FR of the population showed an inverse pattern compared to correct trials (black line). Overall, the normalized FR separated for correct and error trials formed an X-shaped pattern This neural pattern has already been described [5, 7] as a signature of decision confidence. However we are interpreting it here as the neural correlate of difficulty given the lack of a direct measure of confidence in the current task.

### Neural mechanisms for difficulty representation

**Fig 4:**
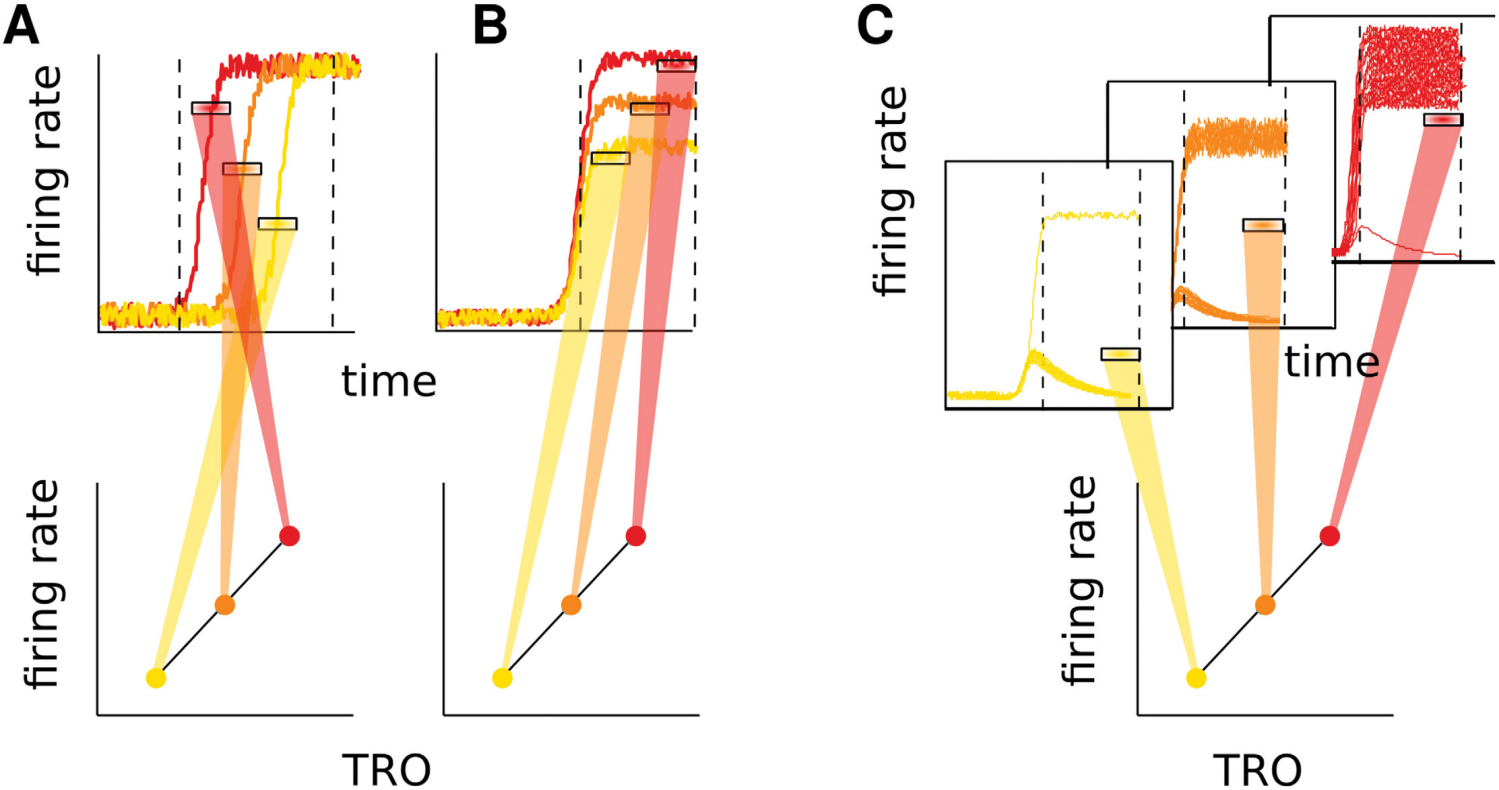
Pictorial representation of possible mechanisms underlying the correlation with difficulty. The figure only illustrates a linear relation between FR and TRO. Panel A: Switch timing code. In the upper panel the time evolution of the FR is shown for three trials (one for each TRO; the color code is the same of the bottom panel). Each trial presents a switch from a state of low activity to a state of high activity. The three horizontal marks show the time averaged FRs taken in the window enclosed in the vertically dashed lines. In the bottom panel the average FRs are shown as a function of TRO. The different switch times of the trials produce different FRs. Panel B: Rate code; each trial reaches a different level of FR in the high activity state (upper panel) and this is reflected in the mean FR (bottom panel). Panel C: Binary code; only some trials switch to the high activity state while others remain in the down state. The number of trials that switch states depends on TRO. When many trials are in an up state (red trials) the mean-over-trials of the time averaged FR is higher in respect to the case of many of the trials in the down state (yellow trials).

The V-shaped pattern shown above can arise from very different computational schemes. In the following, we will try to shed light on this matter. Again only correct trials will be analysed, although the proposed method could easily be applied to error trials. The increasing FR as a function of the absolute value of TRO, i.e., the V-shaped pattern, can arise from at least three distinct mechanisms (for a pictorial representation see Fig 4). 1) Switch time coding (panel A): Neurons increase the FR, switching from a low to a high activity state, with a different timing according to the discriminability, and with the average rate reflecting this timing. 2) Rate coding (panel B): Neurons increase the FR relative to the baseline in proportion to the discriminability. 3) Binary coding (panel C): Neurons have a binary response, i.e., they increase the FR with a probability that depends on the discriminability (e.g. in easy trials the activity is mainly high whilst in difficult trials the neuron mostly remains in a “down” state). In this last scenario mixing trials of high and low activity produces the V-shaped pattern of average FRs (a similar mechanism has been proposed for confidence encoding [21]).

In order to identify neurons implementing each of these mechanisms we used different statistical techniques. Although we present them here as separated mechanisms, we do not rule out the possibility that they could all appear at the same time.

**Fig 5:**
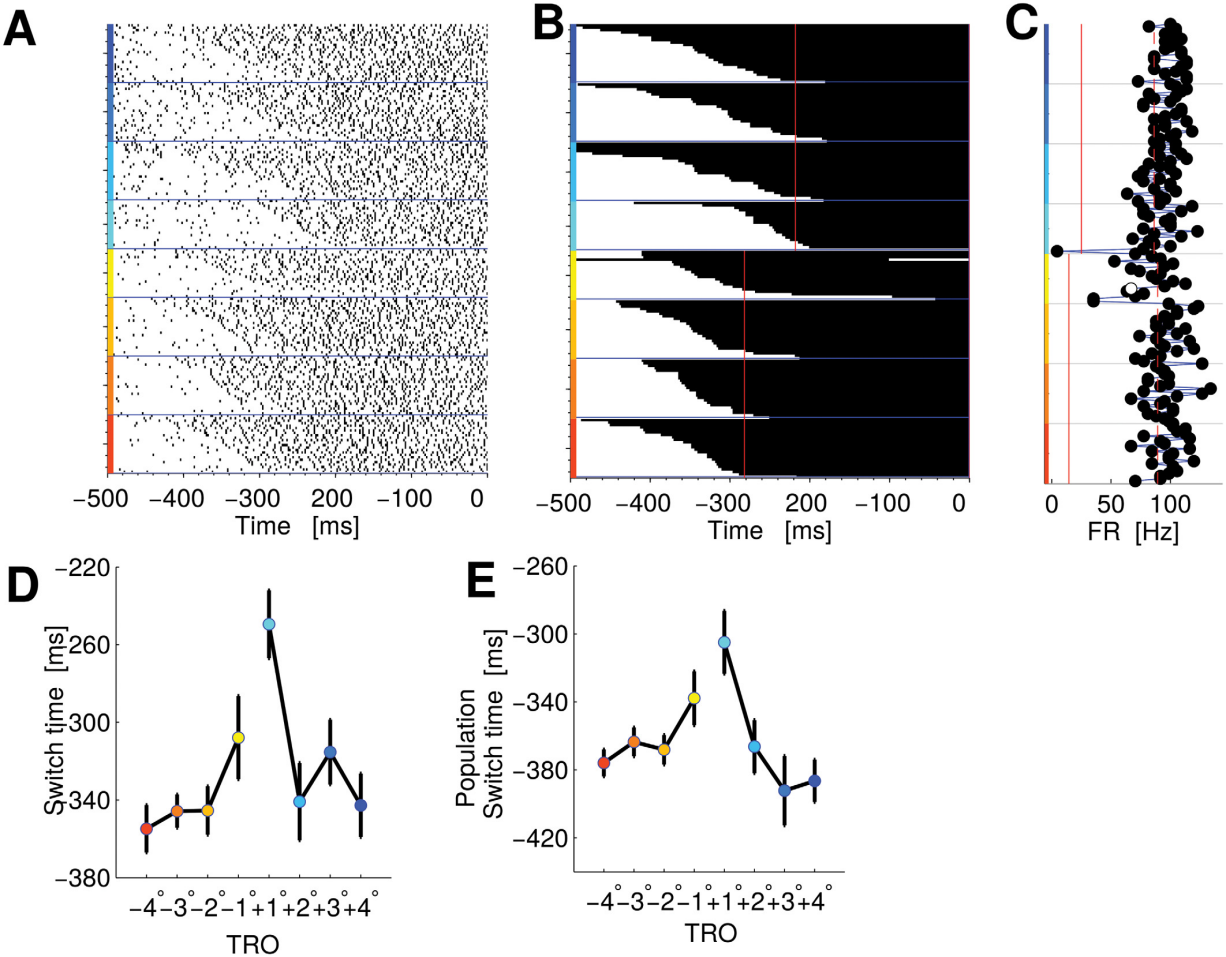
PMv single neuron and population encoding difficulty in the FRs with a switch time code. Panel A: Raster plot. The trials are sorted by the TRO and according to the timing of the state switch, as indicated by the HMM of panel B. Trials for this panel and panel B are aligned to the saccade; around -510 ms the second bar was shown. B: Time course of state switches, according to HMM for the same neuron of panel A. Every row represents a trial. State one is represented by the color white, while black represents state two. Trials start in state one and later change to state two (indicating that they have increased their FR). The vertical red lines indicate when the 90% of the trials have changed state. Panel C: Average FRs of single trials from when the 90% of trials changed state until the end of the trial. The vertical dashed and continuous red lines indicate the average value of FR for high and low state of the HMM analysis respectively. Panel D: Average switch time for this same neuron versus TRO. Panel E: Average switch time for the whole populations of neurons encoding difficulty in the FRs with a switch time code. The color code for the TRO is the same as Fig 1. Error bars of panels D and E represent SEM.

We first verified whether the switch timing had any relevant effect in our data. To do so we used a Hidden Markov Model (HMM) (for its application with single neuron recordings see [26]) which is able to detect when a system switches from one state of activity to another (see Methods for details). In Fig 5 the analysis of a PMv single neuron is shown whose FR encodes difficulty with a switch time code. In Fig 5B we show a summary of the two-state HMM analysis for a single neuron (each row represents a trial). The color of the row changes from white to black when the neuron goes from a low to a high-activity state.

The separation of the two states is clearly visible also in the raster plot (Fig 5A, trials sorted according to the switch time estimated by HMM) and in the time averaged FR of single trials (Fig 5C). This neuron exhibits a lot of variability in the switch timing, changing state from just a few milliseconds up to 300 ms after stimulus onset. The timing of the change was correlated with the difficulty of the trial (Kendall correlation coefficient *τ* = 0.18, *p* < 0.05). Fig 5D represents the mean switch time as a function of TRO. Overall eight neurons showed a significant correlation (*Q* = 0.05) between the switch timing and the difficulty. Fig 5E represents the population average switch time as a function of TRO.

**Fig 6:**
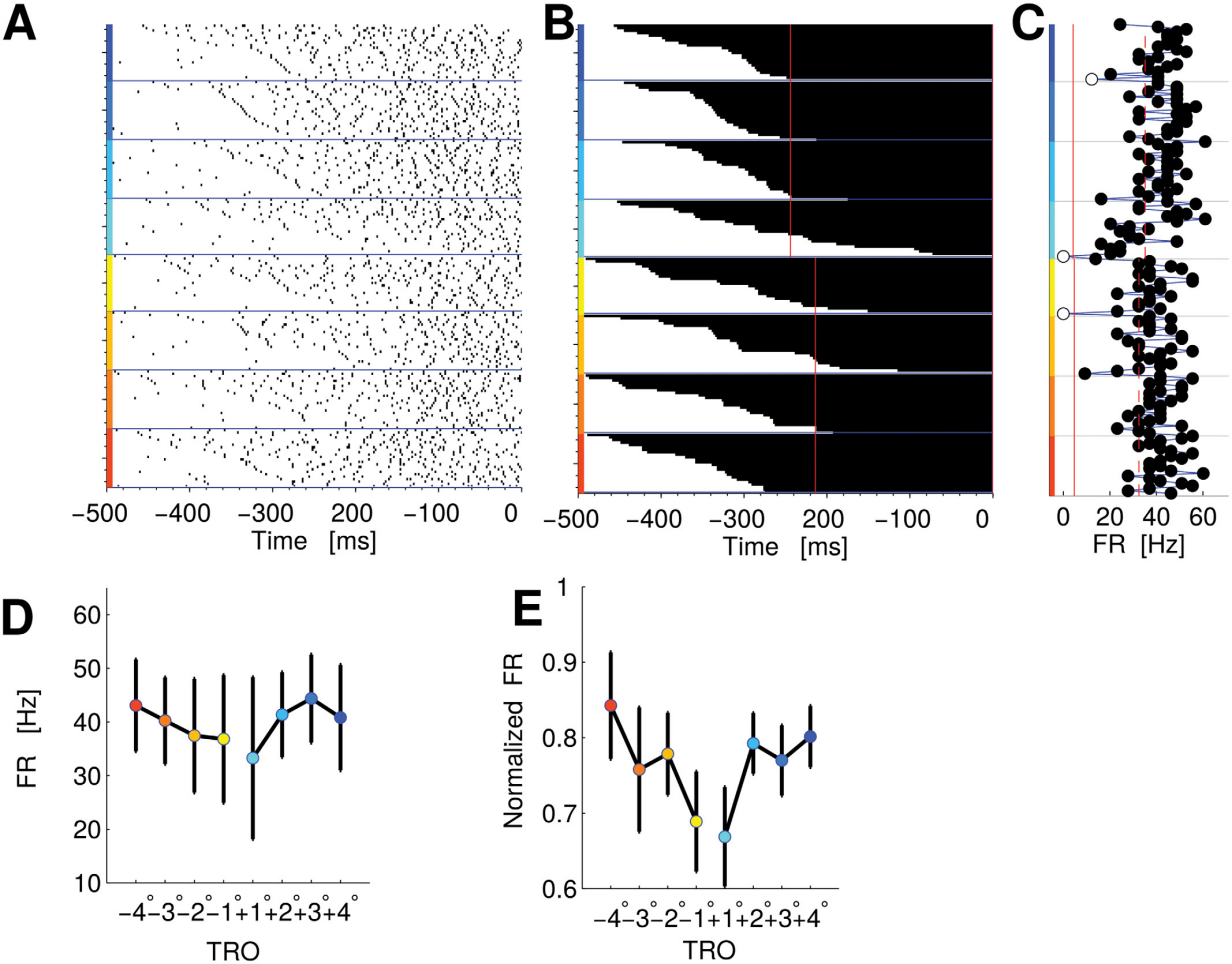
PMv single neuron and population encoding difficulty in the FRs with a rate code. Panel A: Raster plot as in Fig 5A. Panel B: Time course of state switchings, according to HMM for the same neuron of panel A (as in Fig 5B). Panel C: Average FRs of single trials from when the 90% of trials changed state until the end of the trial (as in Fig 5C). Panel D: Average FR for this same neuron versus TRO. Panel E: Normalized average FR for the whole populations of neurons encoding difficulty in the FRs with a rate code. The color code for the TRO is the same as Fig 1. Error bars of panels D and E represent SEM.

Once we had determined when a neuron changes its state we were then able to assess the relevance of the rate coding mechanism. In Fig 6 we show a PMv single neuron whose FR encodes difficulty with a rate code. To estimate whether the increase in FRs was proportional to the difficulty (i.e., the rate coding mechanism of Fig 4B), we first calculated the average FR from when 90% of the trials switched states (red vertical line in Fig 6B), until the coefficient of *LM*_*diff*_ had a significant value (according to the analysis explained above). Then we effectuated a correlation analysis between the level of difficulty and the average FR.

We obtained a significant correlation coefficient for the neuron in Fig 6 (*τ* = 0.24, Kendall correlation, *p* < 0.05), which suggests that it could be the FR of the neuron in the “up” state that encodes the trial’s level of difficulty. The FR of the neuron as a function of TRO is shown in Fig 6D. Overall eighteen neurons had a significant correlation (*Q* = 0.05) between the FR and the difficulty (Fig 6E shows the population average FR as a function of TRO).

To summarize, we found that eight neurons presented a significant impact on the timing in the formation of the pattern, while eighteen neurons increased the FR proportionally to the difficulty of the trial, thereby implementing the rate coding mechanism. There were also twelve neurons that presented both switch timing code and rate code (see Fig 8 for a graphical representation of all classes of neurons). We note that we could apply this method based on the HMM only to 56 neurons of the population (101 neurons) encoding the difficulty, as we considered the HMM analysis was only reliable under certain constraints (see Methods).

Both classes of neurons (that implementing a switch timing mechanisms and that implementing a rate coding mechanism) can be interpreted as continuously encoding the discriminability.

Conversely, the binary mechanism postulated above corresponds to a discrete encoding. Although we may expect a continuous representation, a discretization stage would be needed in order to take subsequent decisions based on the difficulty of a previous one.

**Fig 7:**
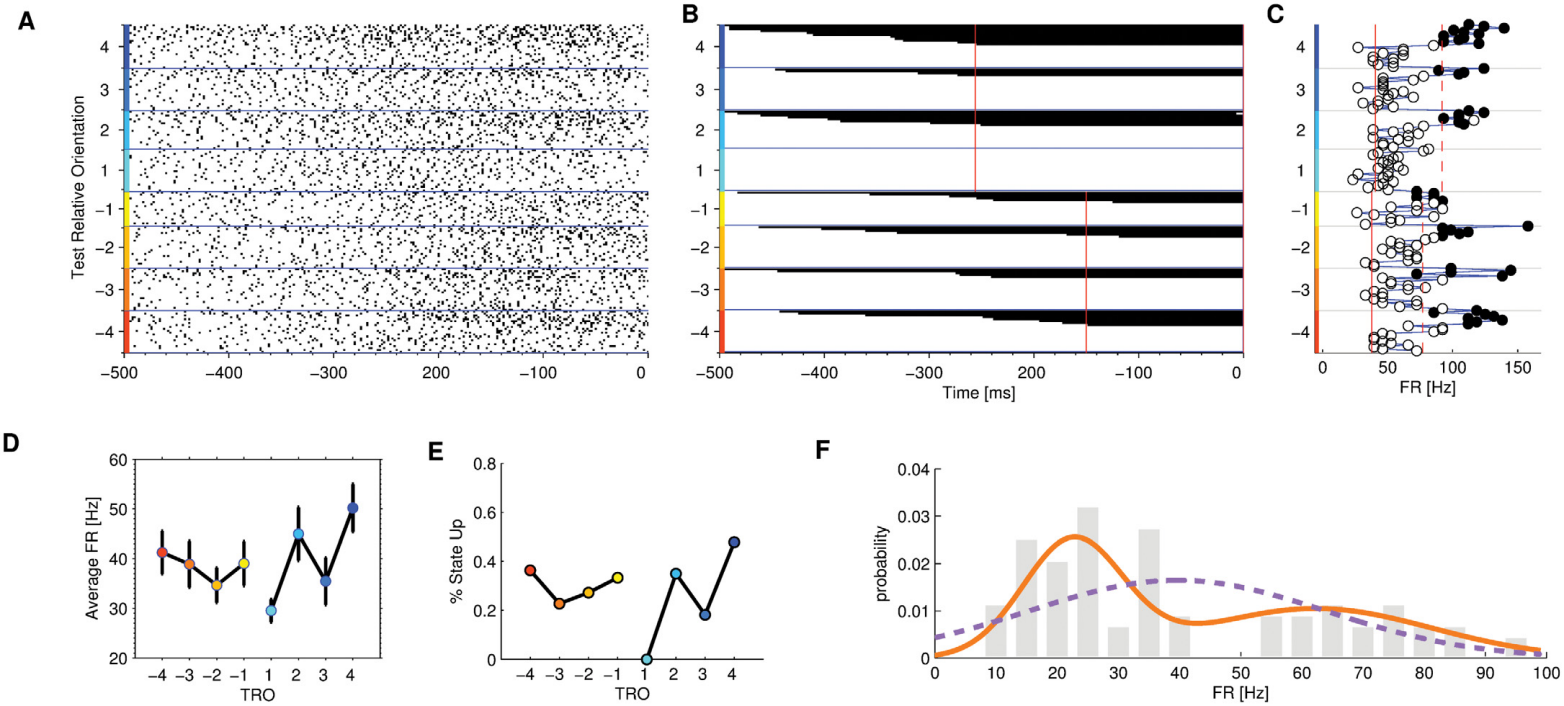
PMv single neuron encoding difficulty in the FRs with a binary code. Panel A: Raster plot as in Fig 5A. Panel B: Time course of state switches, according to HMM for the same neuron of panel A (as in Fig 5B). Panel C: Average FRs of single trials from when the 90% of trials changed state until the end of the trial (as in Fig 5C). Panel D: Average FR for this same neuron versus TRO. Panel E: Probability to be in the high state for this same neuron versus TRO. The color code for the TRO is the same as Fig 1. Error bars of panels D and E represent SEM. Panel F: empirical distribution of mean FR (gray bars), the fitted single Gaussian probability distribution function (dashed purple line) and the Gaussian mixture distribution function (solid orange line).

An example of single neuron implementing the discrete mechanism (identified by the analysis detailed below) is shown in Fig 7.

Fig 7A shows the raster plot, where trials were ordered according to the time of state switch estimated by HMM analysis. We reasoned that the neurons showing a binary behavior should also lead to a characteristic pattern showing up in the HMM analysis: they should present a state switch only on a subset of trials. And indeed this pattern can be seen in Fig 7B (the pattern is only barely observable in the raster plot when trials are ordered according to HMM analysis, Fig 7A). The FR of the neuron after the state switch, as in Fig 7C, also shows a clear separation between the two states identified by HMM analysis. Comparing this figure with Figs 5C and 6C we can clearly see that this neuron switched state only in a subset of trials. The average FR of the neuron is shown in Fig 7D. The FR of the two states does not encode the difficulty of the task and the increasing activity as a function of the absolute value of TRO is due to the increasing proportion of trials in the high activity state as postulated by the mechanism represented in Fig 4C. The proportion of trials in the “high” state is shown for this neuron in Fig 7E.

In order to identify neurons with a discrete response we hypothesized that the distribution over trials of the mean FR as calculated during the test-bar presentation, has to consist of two different distributions. Note that the resulting distribution is not necessarily bimodal but it should differ substantially from the expected Poisson distribution [27–29]. For each trial and each neuron, therefore, we took the average FR in the time-window where neuron was found to be encoding the difficulty (according to the linear model analysis explained above and in the Methods section). S8 Fig shows the encoding window for each neuron. Then we fitted these mean FRs to a Gaussian mixture with two components. The resulting model is the following:

$$\text{GM}=0.5\frac{1}{\sigma_{1}\sqrt{2\pi }}e^{-\frac{{\left(x-\mu_{1}\right)}^{2}}{2\sigma_{1}^{2}}}+0.5\frac{1}{\sigma_{2}\sqrt{2\pi }}e^{-\frac{{\left(x-\mu_{2}\right)}^{2}}{2\sigma_{2}^{2}}},$$

where and *μ*_1, 2_ are the means of the each component and *σ*_1, 2_ the standard deviations, hence the model has four free parameters. We used an expectation maximization algorithm to obtain a maximum likelihood estimator of the parameters of the model (see Methods for details).

In order to rule out the possibility that a single Gaussian distribution model could fit the data better than the mixture model we used the Bayesian Information Criterion (BIC) that, while comparing the likelihood function of the two models, corrects the result by penalizing for the number of free parameters. Therefore, even if the likelihood of the single distribution model were equal to that of the mixture model, the BIC would always prefer the simpler model (or, conversely, a mixture model would be preferable only if it was able to explain much more than the single distribution model).

**Fig 8:**
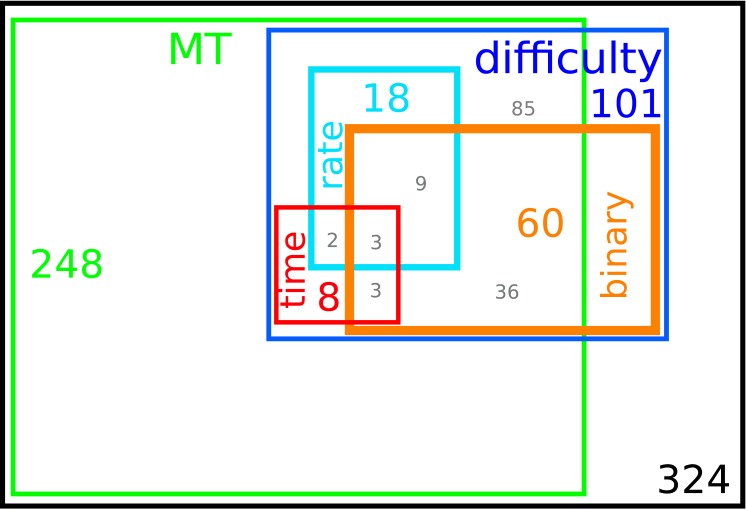
Graphical representation of the different classes of neurons. The colored label and number in each rectangle indicate respectively the class (neurons encoding MT, neurons encoding the difficulty, neurons using a rate code, switch timing code and binary code) and the number of neurons in that set. Gray numbers indicate the number of neurons in the intersections, e.g. twelve neurons are in the intersection between the rate and binary populations and three neurons use all three mechanisms (rate, switching time and binary). Out of the 101 difficulty neurons only 56 could be analyzed with HMM, as explained in the text.

In conclusion, we consider a neuron to have a binary response only if the BIC was giving preference to the mixture model. In addition, to avoid cases where a small difference in the BIC score could favor the mixture model we discarded all those differences that were non significant under the null-hypothesis (H_0_: single Gaussian distribution, FDR controlled with *Q* = 0.05; see Methods for details). We found that sixty neurons displayed a binary mechanism in the case of at least one behavioral response (e.g., “left”) and seventeen of those for both behavioral responses (see Fig 8 for a graphical representation of all classes of neurons). In these neurons the V-shaped pattern of the FR can arise because the proportion of trials with high FR correlates with the difficulty of the trial.

In Fig 8 we show the different classes of neurons (neurons encoding MT, neurons encoding the difficulty, neurons using a rate code, switch timing code and binary code). The label and number in each rectangle indicate respectively the class and the number of neurons we found in that set. We also report in gray the number of neurons in the partial intersections, e.g. five neurons are in the intersection between the rate and switch time populations and three neurons use all three mechanisms (rate, time and binary).

## Discussion

In this study we tackled the following question: What are the mechanisms in the primate brain that encode the difficulty of a decision in single trials?

We have shown that the MT correlates with the discriminability of the stimulus and hence it could be used as a cue to infer the difficulty of a trial. The correlation of RT and difficulty has been proven by many experimental studies [15–17]. Theoretical studies [18, 19] suggest that this correlation is attributable to the decision time (more than movement time or sensory processing time). As in our task the subject is not allowed to respond until the end of the second stimulus, we were only able to record the MT. The decision process of the subject could in principle extend over the duration of the second stimulus, therefore, the MT could embrace part of the decision time and part of the motor preparation. In addition we cannot exclude a correlation between the time for motor execution and the difficulty. Our result is particularly important since it opens new questions about the distinct functions of decision time and MT in the formation of the RT. Moreover it shows that the common choice to model non-decision time as a fixed quantity [17, 19, 30, 31] could be not appropriate depending on the purposes of the model. In addition we found that neurons activity in PMv is informative about the MT. Moreover we have demonstrated that the FR of neurons in primate PMv encode stimulus discriminability.

The variability of neural responses could be explained by different computations performed by neurons in single trials that, once averaged, could produce the same pattern. We suggested three hypothesis for these computational mechanisms: 1) The switch time coding: when the activity of the neuron changes, the difficulty of the decision, is encoded in the timing of the change, 2) The rate coding: the difficulty is encoded in the FR, after the change has taken place; or 3) The binary coding: the neuron only switches between a high and a low activity state and the proportion of high activity trials depends on the discriminability of the stimulus. The first two alternatives correspond to a continuous encoding of difficulty, whereas the last one is a form of discrete encoding. We found, in fact, evidence for all three mechanisms in monkey PMv neurons. For certain neurons the timing and FR mechanisms work together, i.e., a neuron that changed state earlier on less difficult trials will also have a higher FR after the change. Other neurons present a binary response (increasing activity only in some trials), which suggests a possible role in more complex decision scenarios where decisions must be taken based on the difficulty of previous ones.

An important question is: why should neurons use only one mechanism to encode difficulty? Our hypothesis is that difficulty neurons carry-out more than one function in the sensory-motor path. It seems natural that difficulty may be encoded on a continuous scale, since we usually think about difficulty as a graded quantity. However, if difficulty is to have behavioral relevance, then, depending on the requested output, the information about difficulty may need to be discretized. Our hypothesis is that, while certain neurons use a continuous representation, other neurons read-out this scale and transform it into a discrete quantity in order to produce consistent behavior.

This hypothesis highlights the fact that all three proposed encoding mechanisms are not only evidenced by our decoding procedures but they stand as natural representations of difficulty that can easily be read out by higher processing brain areas. Indeed if a read out neuron could have access to the distribution of FR or to that of switch timing, this neuron could measure the difficulty of the decision. Binary neuron could implement for example a type of read out process where the difficulty information encoded by FR of switch timing statistics is used to give a binary classification of difficulty.

Most of our results depend on a linear model of the FR. But does this relation have to be linear (and not, for example, logarithmic or sigmoidal)? Firstly we note that linear functions have been extensively used to model the relation between the FRs of neurons and certain task features (e.g. [4, 32, 33]). Yet it is possible for the relation not to be linear. Indeed, we consider the linear function as a first probable approximation.

In order to assess the reliability of the linear model we also analyzed the R^2^ of the linear model and considered a neuron to carry information about the difficulty only if the R^2^ was significantly higher than chance (see Methods for details). Using the MT as a regressor for the linear model 90% of neurons with significant slope of the regression line showed significant values of the R^2^. Using the difficulty of the task as regressor, 61% of neurons with significant slope of the regression line had also significant values of R^2^ meaning that the linear model is able to explain enough variance of most neurons. However the remaining 39% of neurons had lower values of R^2^ suggesting that non-linear methods could maybe explain better those data. In general the applied method suggests that simple assessment of the statistical significance of the slope of regression line could be a weak control in this type of analysis and that the R^2^ can provide useful insight into the goodness of the linear model. We provide a non parametric test to address how much the R^2^ values are different from those produced by chance results.

The three mechanisms underlying the difficulty V-shaped pattern that we have suggested, raise the question of whether PMv neurons change their FR gradually, or whether they jump from a low to a high activity state. This question, that has often raised concerning the decision neurons of the lateral intraparietal sulcus (LIP), has been bothering the scientific community for some time now [3, 15, 34, 35]. Recently, [36] reliable evidence has been provided for the hypothesis that LIP neurons display a gradual ramp. Although our analysis was aimed at differentiating single trial mechanisms, we did not address this issue. We do note that all three proposed mechanisms are compatible with both a gradual and an abrupt transition of states.

Our results could also suggest another interpretation: that the PMv neurons are actually encoding the confidence or uncertainty in the decision. There is a growing body of research on the role of uncertainty estimation in perceptual decisions, on its neural representations and on its computational substrates [7, 9, 21, 24, 37, 38]. The difficulty of a decision is one of the main factors influencing the confidence in that decision as demonstrated by many experimental [20, 24, 39, 40] and theoretical [20, 21, 38, 41] studies. In our experimental setup the stimuli were well visible, hence the perceptual uncertainty of the stimuli was reduced compared to other situation (e.g. when the temporal or spatial integration of the signal is necessary to reduce uncertainty in the estimation of the relevant variable: random dots motion direction discrimination, numerosity estimation, etc.) and the uncertainty in the decision was mainly affected by the relative difference in orientation of test and reference bar. Confidence measures are related to the (objective) difficulty of task with a typical X-shaped pattern: The positive correlation with discriminability in correct trials is mirrored (i.e. negative correlation) in error trials [22]. The X-pattern may suggest a role of these neurons in metacognitive processing, nonetheless we note that this pattern is neither necessary (Kornell, 2013, Kornell et al., 2011, Kiani et al., 2014) nor sufficient to define confidence, since it could also emerge from different computations (Insabato et al. in preparation). Indeed when we represent the MT as a function of discriminability and separated for correct and error trials it shows the typical X-pattern (Fig 1C). It is indeed likely that the MT correlates with confidence, since it is well known that the decision time is related to decision uncertainty [20, 38, 42]. If the behaviour of subjects could then reflect the uncertainty or confidence in the decision, this may also be present in the neural recordings. Moreover the population FR of integrative neurons separated for correct and error trials formed the X-shaped pattern (Fig 3D). We cannot rule out that the population of integrative neurons is encoding the confidence in the decision and not only the difficulty. If this were true the proposed encoding mechanism for difficulty could actually serve as mechanisms for uncertainty coding. We could speculate that the continuous coding schemes proposed may serve as a representation for decision uncertainty, while the binary mechanism may form the basis of a classification of uncertainty for confidence rating or confidence guided behaviour. However the task we used has no direct confidence measurement and therefore reasonable doubts could be cast on this interpretation of the results.

Although our results do not directly support the interpretation of a neural representation of decision confidence in PMv, they demonstrate neurons in PMv involved in the encoding of the difficulty, which is a building-block for the construction of confidence.

## Methods

### The discrimination task

Experiments were made using two male monkeys (Macaca mulatta). Animals (BM5, 8 kg; and BM6, 6 kg) were handled according to the standards of the European Union (86/609/EU), Spain (RD 1201/2005), and the Society for Neuroscience Policies and Use of Animals and Humans in Neuroscience Research. The experimental procedures were approved by the Bioethics Commission of the University of Santiago de Compostela (Spain).

The monkeys’ heads were immobilized during the task and looked binocularly at a monitor screen placed 114 cm away from their eyes (1 cm subtended 0.5 to the eye). The room was isolated and soundproofed. Two circles (1° in diameter) were horizontally displayed 6° at the right and 6° at the left of the fixation point (a vertical line; 0.5° length, 0.02° wide) displayed in the screen center. The monkeys used right and left circles to signal with an eye movement the orientation of visual stimuli to the right and to the left, respectively. Orientation Discriminations Task: the monkeys were trained to discriminate up to their psychophysical thresholds in the visual discrimination task sketched in Fig 1A (training lasted for approx. 11 months). The stimuli were presented in the center of the monitor screen and eye movements larger than 2.5° aborted the task. The orientation discrimination task was a two-interval, two-alternative forced-choice task. A masking white noise signaled the beginning of the trial and then the fixation target (FT) appeared in the center of the screen (Fig 1A). The monkey was required to fixate the FT. If fixation was maintained for 100 ms, the FT disappeared, and, after a variable pre-stimulus delay (100–300 ms), two stimuli (S1 and S2), each of 500 ms duration, were presented in sequence, with a fixed inter-stimulus interval (1 s). At the end of the second stimulus, the subject made a saccadic eye movement, in a 1200 ms time window, to one of the two circles, indicating whether the orientation of the second stimulus was clockwise or counterclockwise to the first. We also recorded the movement time (MT) of the subject, the time from the end of the second interval (S2) until the response. The orientation of the test bar relative to the reference (test relative orientation, TRO = *S*2 − *S*1) manipulated the difficulty of the task. Trials lasted approx. 3.5 s separated by a variable intertrial interval (1.5–3 s). Fifty milliseconds after the correct response, a drop of liquid was delivered as a reward. A modulation of the masking noise signaled the errors; the modulation started 50 ms after the incorrect response and lasted for 75 ms.

Monkeys’ weights were measured daily to control hydration, and once a week the animals had access to water ad libitum. The level of training was assessed by the psychometric functions. Once trained, the monkeys performed around 1000 trials per day. The lines were stationary, subtending 8° length and 0.15° wide. Three different S1 orientations were used for each monkey during the recordings: 87°, 90°, and 93° (BM5) and 84°, 90° and 96° (BM6); all angles referred to the horizontal axis. Different S2, eight per S1, were presented, four clockwise and four counterclockwise to S1 in steps of 1° (BM5) and 2° (BM6). More details can be found in [4].

### Recordings

Neuronal population: extracellular single-unit activity was recorded with tungsten micro-electrodes (epoxylite insulation, 1.5-3.5M, catalog # UEWMGCLMDNNF; FHC) in the posterior bank of the ventral arm of the sulcus arcuatus and adjacent surface in the ventral premotor cortex in the four hemispheres of the two monkeys (see [4], for a detailed description of the recording sites). In this work, we studied the responses of a subset (324) of the recorded neurons. This subset was selected with a ROC analysis of FR with respect to the choice (see [4] for details).

### Data analysis

All analyses were performed using custom-made programs in Matlab. Unless noted otherwise, statistical analyses were applied to the FRs of single neurons during the 500 ms preceding the saccade. In fact, the second stimulus was presented during this period, and therefore the decision-making process was expected to take place during this time window. Our aim was to find any existing neurons whose activity relates to:

1. Movement Time (MT): Neurons whose mean FR increases or decreases linearly with the MT.
2. Discriminability of the stimulus: Neurons whose mean FR for the correct trials increases or decreases linearly with the difficulty.

In order to accomplish this we used a linear regression analysis [43]. Of course, linearity is only one of numerous possible encoding mechanisms, even when we take only those concerning FRs into consideration. We decided on this for the sake of simplicity.

As experiments were done using animals that were awake it was very difficult to record single neurons over a long period, therefore the number of error trials for each neuron was very low and error trials were excluded from the linear analysis of difficulty as noted below.

### Linear analysis

The FR of the last 500 ms before the saccade was computed by averaging the spike count in a sliding window of 100 ms slided with a step of 20 ms. In this way we got for each trial and each neuron a time series *r*(*t*) of the FR, where t is time discretized in 25 time bins.

To individuate the neurons presenting a modulation of the movement time the following linear model (*LM*_*mt*_) analysis was used *r*(*t*) = *d*_1_(*t*) *MT* + *d*_2_(*t*), where *d*_1_, *d*_2_ are the parameters to be fitted. In order to assess the significance of the coefficients a Monte-Carlo random resampling method was used. This method allows to estimate the distribution of the parameters under the null hypothesis (no dependence between the FR and the MT). To this aim we built 100 surrogated data sets by randomly reassigning the labels (MT values), thus each surrogate was constructed by permuting the values of MT over all trials. This randomization destroys eventual correlation between the FR and the MT. We then applied the *LM*_*mt*_ to each surrogate and obtained the estimated distribution of the coefficients under the null hypothesis. Neural activity in a bin *t* was considered linearly dependent on the MT if the coefficient *d*_1_(*t*) had a low probability (*p* < 0.05) under the null hypothesis. In addition we required a minimum number of consecutive significant bins in order to avoid false positive results due to fluctuations. The minimum number of significant bins was chosen by estimating the probability distribution of N consecutive significant bins under the null hypothesis. We used again a Monte-Carlo random resampling method to estimate this probability distribution. For each surrogate we marked all the bins with a low probability under the null hypothesis (*p* < 0.05) and extracted the maximum number of those bins that were consecutive. This procedure gave us an estimate of the number of consecutive significant bins under the null hypothesis. We considered the activity of a neuron to be dependent on the MT if the maximum number of consecutive significant bins had a low probability under the null hypothesis. In order to correct for multiple comparison (we applied the same test to all neurons) we used the Benjamini-Hochberg procedure [25] to control the False Discovery Rate to a value *Q*. We show in S1 Fig the results of using different values of *Q*. The number of total discoveries is represented by bars (errorbars represent standard deviation of bootstrapped data) as a function of the value of *Q*. The dashed red line represent the maximum number of accepted false discoveries. We can observe that there are many true discoveries in the set of accepted discoveries, indeed the number of total discoveries is much higher than the maximum number of accepted false discoveries for a wide range of *Q*. This shows that our findings are robust over a wide range of *Q*. However to further analyse the neurons found to be significant, e.g. to calculate the average population activity or to show the activity of one single neuron, we used *Q* = 0.05 in order to keep the number of false discoveries low. To give more insight on this method, appendix S1 Text presents a description of Benjamini-Hochberg procedure [25] for varying *Q* in the analysis of a synthetic dataset, where the ground truth is known.

It is possible that the slope of the regression line, *d*_1_, is significantly different than that obtained by chance under the null hypothesis but still the *LM*_*mt*_ is explaining a small part of the variance of data. In order to assess the portion of explained variance we calculated the coefficient of determination (R^2^) of the *LM*_*mt*_. The values of the R^2^ were in the range between 0 and 0.75. To determine which values of the R^2^ were high enough, we estimated the distribution of the R^2^ under the null hypothesis, with the same Monte-Carlo random resampling methods explained above for *d*_1_, and applied the same constraints as above (p-value, minimum number of consecutive bins, multiple comparison correction). Finally we considered a neuron to be informative about the MT if both R^2^ and *d*_1_ were significant (FDR controlled with Benjamini-Hochberg procedure) in the same interval. This procedure was used for all linear models used in this study.

To individuate the neurons presenting a modulation of task difficulty only correct trials were used and the following linear model (*LM*_*tro*_) analysis was used independently on trials with positive and negative values of TRO: *r*(*t*) = *d*_1_(*t*) *TRO* + *d*_2_(*t*), where *d*_1_, *d*_2_ are the parameters to be fitted. The significance of the coefficients was assessed with a Monte-Carlo random resampling method was used as explained above for the *LM*_*mt*_. In addition a minimum number of consecutive significant bins was required (as explained above for the *LM*_*mt*_) in order to avoid false positive results due to fluctuations. Finally only neurons with significant R^2^ were considered to encode TRO, similar to the above explained analysis of *LM*_*mt*_. In S2 and S3 Figs, both for the R^2^ and for the slope of the linear model, we show the resulting number of discoveries as a function of *Q* (same conventions as in S1 Fig). It is easy to observe, in both figures, that in a wide range of *Q* the number of total discoveries is much higher than the maximum number of accepted false discoveries. For further analysis on this group of neurons we used *Q* = 0.1. Moreover we want to emphasize that the difference between the number of total discoveries and the maximum number of accepted false discoveries (i.e. the distance between bars height and the dashed red line) first increases, then stabilizes in a wide range of *Q* and then decreases towards *Q* = 1. For example, in S3 Fig, for *Q* = 0.1 44 total discoveries are made and 4 of them are expected to be false, hence 40 discoveries are true; for *Q* = 0.2, 88 total discoveries are made and 18 of them are expected to be false, hence 70 discoveries are true. In general, under these conditions, if one want to find more true discoveries (more power) a higher Q-value may be chosen, e.g. *Q* = 0.2, although one must be always aware that this higher number of true discoveries comes at the price of more false discoveries.

These neurons are not necessarily encoding TRO for both choices of the subject (left or right) since the *LM*_*tro*_ was fitted separately for positive and negative values of TRO. However the whole ensemble of neurons encodes the information about the TRO. Then we looked for neurons, that can integrate the information encoded by this ensemble and represent the difficulty of the trial. Such neurons would present a V-pattern when the FR is plotted as a function of TRO (or a reversed V). In order to find this integrative difficulty neurons the following linear model (*LM*_*diff*_) was used *r*(*t*) = *d*_1_(*t*) ∣*TRO*∣ + *d*_2_(*t*), where *d*_1_, *d*_2_ are the parameters to be fitted. The same statistical testing procedure was used as for the other linear models in order to assess the significance of results. In S4 Fig we show the total number of discoveries as a function of Q-value as for the other models described above. Here again we observe that our results are robust over a wide range of *Q*. However for further analysis on this group of neurons we used *Q* = 0.1.

In order to produce Fig 3D the FR of each neuron was normalized to its maximum value and then the activity of all neurons was averaged together.

### Mechanism for the difficulty neurons

We individuated three possible neural mechanisms responsible for the above mentioned modulation of the difficulty neurons. A simplified representation of these mechanisms is presented in Fig 4. In order to understand which difficulty neuron belongs to each of the three categories, we applied different methods:

1. In order to find neurons that switch states with a timing dependent on the difficulty, we used the Hidden Markov Model (HMM) analysis [44] to estimate the time of neural activity change due to the test bar. Indeed, the HMM was able to cluster the spiking activity of individual neurons into periods of ‘stationary’ activity (the states) within a single trial. Hence the switch time between states could be estimated.
2. In order to find neurons whose activity after the change, as estimated by HMM, encoded the difficulty, we calculated the correlation between the mean activity and the difficulty of the task. The mean activity was calculated in the time window starting at the time bin where the 90% of the trials had passed from one state to the other and ending at the last significant time bin marked by the *LM*_*diff*_ or *LM*_*tro*_.
3. In order to find the neurons whose activity could be explained as a compound of high and low FR states we fitted (with Expectation Maximization algorithm) the FR distribution to a Gaussian mixture model.

For each one of this method we controlled the FDR with Benjamini-Hochberg procedure [25]. Also in this case the number of true discoveries was quite stable over a wide range of *Q*; the reported results are for *Q* = 0.05.

### Hidden Markov model

To analyze the single-trial activity of the recorded neurons we used the Hidden Markov Model that clusters the spiking activity of individual neurons into periods of stationary activity within a single trial. The HMM technique has been successfully applied to characterize the single-trial activity of cortical neuronal ensembles during movement with holding and preparation [45, 46], taste processing [47], and perceptual decision making [3]. Here, we briefly review some aspects of the HMM analysis; more details about the algorithms can be found in previous works [3, 45, 47].

Within the HMM, the activity of a recorded neuron at time *t* is assumed to be in one of a (predetermined) number (*Q*) of hidden FR states. In each state q, the discharge of a neuron is assumed to be a Poisson process of intensity *λ*_*q*_, which defines the instantaneous firing probability *E*_*q*_, i.e., the probability of firing a spike within one time bin, equal to 2ms throughout this study. States are said to be hidden because they are not directly measured; instead, we observe the stochastic realizations of the state-dependent Poisson process (observation sequences). The state variable changes from state *i* to state *j* with fixed probabilities that defined a transition matrix *A*, given by *A*_*ij*_ = *P*(*q*_*t*+1_ = *j*∣*q*_*t*_ = *i*), where *q*_*t*_ is the state at time t and *i*, *j* ∈ {1, …, *Q*}. The entire process is a Markov chain: the transition probabilities *A*_*ij*_ are independent of time, i.e., they depend only on the identities of states *i* and *j*, which means that the state sequence at time t only depends on the state at time *t* − 1. In summary, for a single neuron the HMM is fully characterized by the spike-emission probabilities (*E*) and the transition matrix (*A*). These model parameters are estimated from the data, using a likelihood expectation-maximization algorithm [3, 45, 47].

Briefly explained, the procedure starts with random values for *E* and *A* and re-estimates the parameters to maximize the probability of observing the data given the model. After optimization of the model parameters, the Viterbi algorithm is used to find the most likely sequence of hidden states given, for each single trial, the model and the observation sequence [3]. In the present study we used the HMM to detect the transitions between a state of low and a state of high activity. For this reason, the number of states was set to *Q* = 2. For each neuron, the data was divided into two subsets, composed of trials corresponding to each behavioral response (left or right). For each subset, a HMM was estimated using the activity of 80% of the trials (randomly selected) during the period within the last 500 ms before the saccade. After optimization the most likely state sequence was stored for all trials.

Unfortunately, a HMM analysis was not reliable for all the neurons. We only considered the HMM reliable if a) the mean duration over trial of both states was at least 25 ms. (i.e., we do not take into account states with very brief duration), b) the number of state-switches per trial was three or less; or (i.e., we do not take into account bursting neurons), c) at least five of both the left and right oriented trials had a state-switch (i.e., we want neurons with 2 different states). We found 26 difficulty neurons (out of 101) whose HMM was interpretable. For this subset, we wanted to distinguish between the three V-shaped mechanisms, to do so we analyzed the state-switch time. For each trial the HMM gave the time in which it changed from a low to a high state (or vice versa).

We also performed the analysis with three hidden states (*Q* = 3). After applying the above mentioned constrains, we found only five neurons for which the three states HMM was reliable. For these neurons the third state was associated with the slowly increasing activity, which may be detected as an intermediate state. S5 Fig shows the dynamics of the hidden states for one of the three neurons. Since we are interested only in the timing of the change from inter-stimulus period activity to activity induced by the second stimulus we didn’t take into account the three states HMM. In addition the BIC selects the two states HMM as the better model for all these five neurons.

In S6 Fig we show the time course of state switch for the whole neural population. Overall the population switches from state one to state two gradually over time. However the curves in S6 Fig are not strictly monotonically increasing, meaning that some neuron present a second switch from state two to state one. Indeed we mainly found just one switch per neuron but some neurons presented also a return to state one: they respond rapidly to the test bar stimulus and then go back to a lower activity state. This dynamics is illustrated for an example neuron in S7 Fig.

### Binary neurons

Our aim was to investigate whether the FR distribution of neurons during correct trials was better described using a mixture model composed of two distributions rather than a single distribution. The procedure we applied was the following:

1. For each neuron trials were divided into two sets, depending on their behavioral responses (“left” or “right”). We calculated the average FR for each trial in the encoding window of that neuron. The encoding window was the largest continuous interval of significant bins found by the linear models as described above in the Linear Analysis section (i.e. where both slope and R^2^ were significant). These encoding window are shown in S8 Fig. If a neuron had no encoding window for one of the behavioral responses, the corresponding trials were not taken into account. For each of the two behavioral responses, the longest interval was taken among those found by *LM*_*tro*_ and *LMdiff*.
2. The distributions were fitted with a function *GM*, which was the average of two Gaussian distributions:
  $$\text{GM}=0.5\frac{1}{\sigma_{1}\sqrt{2\pi }}e^{-\frac{{\left(x-\mu_{1}\right)}^{2}}{2\sigma_{1}^{2}}}+0.5\frac{1}{\sigma_{2}\sqrt{2\pi }}e^{-\frac{{\left(x-\mu_{2}\right)}^{2}}{2\sigma_{2}^{2}}},$$
  where and *μ*_1, 2_ are the means of the each component and *σ*_1, 2_ the standard deviations, hence the model has four free parameters. We used an expectation maximization algorithm to obtain a maximum likelihood estimator of the parameters of the model. Given the stochasticity of the optimization procedure, the fitting was repeated 10 times and the best solution in terms of likelihood was retained, in order to avoid local minima.
3. In order to verify whether the mixture model explains the data better than a single distribution model, we fitted the data to a single Gaussian distribution and then we compared the two models using the Bayesian Information Criterion (BIC) [48, 49], which is given by:
  BIC=-2lnL+pln(T)
  where *L* is the maximized value of the likelihood function for the estimated model; *p* the number of free parameters of the model (2 or 4); and *T* the length of the observation data (the number of trials in our case). This means that the BIC method penalized the model likelihood by a measure of its complexity (i.e., the number of free parameters). The single distribution model has two free parameters, while the mixture model has four free parameters. Therefore, in order to have a better score in the BIC, the higher complexity due to the second component should be really well balanced by a better ability to explain the data. Another common choice for a model comparison technique would have been the Akaike Information Criterion. The main difference between the two is in the penalization term due to the number of free parameters of the model and amount of data points, where BIC penalizes more model complexity. Since our aim was to find neurons better described by a mixture of two distribution we chose the BIC in order to have a more conservative method.
4. We took all those neurons for which the BIC score of the mixture distribution (*BIC*_*GM*_) was lower than the BIC score of the single distribution model (*BIC*_*G*_); recall that the lower the BIC the better the model. However it is possible that small differences in BIC scores (Δ*BIC* = *BIC*_*GM*_ − *BIC*_*G*_) produce non-significant results. For this reason, for each those neurons, we calculated the probability of obtaining such a Δ*BIC* under the null-hypotheses and only retained, as binary response neurons, those having significant p-values. The distribution of Δ*BIC* under the null-hypothesis (single component distribution) was built using a Monte-Carlo random resampling technique: For each neuron the parameters of the single Gaussian distribution fitted to the original data set of mean FR, were used to generate a new data set of the same size as the original one; then a single Gaussian and a Gaussian mixture were fitted to the new data set and the BIC score was calculated for each model, and the corresponding Δ*BIC*; this process was repeated 2000 times, resulting in a distribution of Δ*BIC* under the null hypothesis of data generated by a single distribution. Finally, as already explained, we considered a neuron to have a binary response if the p-value of its Δ*BIC* was significant (FDR controlled with Benjamini-Hochberg procedure, *Q* = 0.05).

S9 Fig shows a visual summary of this analysis for a binary neuron, while S10 Fig show the results for a non-binary neuron.

## Supporting Information

S1 Text FDR control with a synthetic dataset. We tested the functioning of the Benjamini-Hochberg procedure to control the FDR, varying *Q*, with synthetic datasets, where the ground truth is known. When no false null-hypotheses is present, no discovery is made over the whole range of *Q*. When a given number of false null-hypotheses is present, the number of true discoveries is never higher than the number of false null-hypotheses over the whole range of *Q*.
(PDF)

S1 Fig Total discoveries of *LM*_*mt*_. Number of total null hypothesis rejected as a function of *Q* value for the linear model *LM*_*mt*_. The number of total discoveries is represented by bars (errorbars represent standard deviation of bootstrapped data) as a function of the value of *Q*. The dashed red line represents the maximum number of accepted false discoveries.
(PDF)

S2 Fig Total discoveries of *LM*_*tro*_ for *TRO* > 0. Number of total null hypothesis rejected as a function of *Q* value for the linear model *LM*_*tro*_ and for positive values of TRO. The number of total discoveries is represented by bars (errorbars represent standard deviation of bootstrapped data) as a function of the value of *Q*. The dashed red line represents the maximum number of accepted false discoveries.
(PDF)

S3 Fig Total discoveries of *LM*_*tro*_ for *TRO* < 0. Number of total null hypothesis rejected as a function of *Q* value for the linear model *LM*_*tro*_ and for negative values of TRO. The number of total discoveries is represented by bars (errorbars represent standard deviation of bootstrapped data) as a function of the value of *Q*. The dashed red line represents the maximum number of accepted false discoveries.
(PDF)

S4 Fig Total discoveries of *LM*_*diff*_. Number of total null hypothesis rejected as a function of *Q* value for the linear model *LM*_*diff*_. The number of total discoveries is represented by bars (errorbars represent standard deviation of bootstrapped data) as a function of the value of *Q*. The dashed red line represents the maximum number of accepted false discoveries.
(PDF)

S5 Fig Three states HMM. Time course of state switches for one neuron showing three hidden states of a HMM. A two states HMM is shown for comparison. Panel A: raster plot. Panel B: dynamics of hidden states of two states HMM. Each row represents a trial. State one is represented by the color white, while black represents state two. Panel C: dynamics of hidden states of three states HMM. Each row represents a trial. State one is represented by the color white, black represents state two and gray represents state three. The third state is associated to the initial phase of ramping activity. According to BIC the two states HMM is a better model than the HMM with three states. There were only five neurons showing a consistent third state and for all of them the BIC favors the two states HMM.
(EPS)

S6 Fig Dynamics of state switches in the neural population. The proportion of trials in state two (“up” state), according to a two states HMM is represented as a function of time. The proportion is over all trials from all neurons. Each line represents trials grouped by TRO, colored according to the color code explained in Fig 1. Positive (upper) and negative (bottom) values of TRO are plotted in separate panels for better visibility. Overall it is visible that the population switches from state one to state two gradually over time.
(EPS)

S7 Fig Single neuron with two state switches. Panel A: Raster plot. The trials are sorted by the TRO and according to the timing of the state switch, as indicated by the HMM of panel B. Trials for this panel and panel B are aligned to the saccade; around -510 ms the second bar was shown. Panel B: Time course of state switches, according to HMM for the same neuron of panel A. Every row represents a trial. State one is represented by the color white, while black represents state two. Trials start in state one, later change to state two (indicating that they have increased their FR) and then return to state the activity level of state one. The vertical red lines indicate when the 90% of the trials have changed state. Panel C: Average FRs of single trials from when the 90% of trials changed state until the end of the trial. The vertical dashed and continuous red lines indicate the average value of FR for high and low state of the HMM analysis respectively.
(EPS)

S8 Fig Encoding window of difficulty neurons. Each row represents a difficulty encoding neuron. For each neuron the time window where the linear model was significant (i.e. both slope and R^2^ were significant, see Methods) is shown. In this encoding time window, colors represent the slope value of the linear model. All other time bins are represented in black. The three panels correspond to the linear model indicated in the respective title. Time is aligned to the saccade.
(PDF)

S9 Fig Binary neurons analysis for a single binary neuron. Each column corresponds to one behavioral response (i.e. *TRO* < 0 or *TRO* > 0). Top row show the empirical distribution of mean FR (gray bars), the fitted single Gaussian probability distribution function (dashed purple line) and the Gaussian mixture distribution function (solid orange line). Middle row show the distribution of Δ*BIC* values under the null-hypothesis (blue bars) and the value of the Δ*BIC* corresponding to the real data set (red square). Bottom row show the average firing rate over time in the time window starting 100 ms before the encoding time window and ending 100 ms after it. The trials were sorted according to the component of the mixture they belong to (i.e. the one for which the posterior probability of the latent variables of the expectation maximization algorithm was higher). Shaded area represent SEM.
(PDF)

S10 Fig Binary neurons analysis for a single non-binary neuron. Each column corresponds to one behavioral response (i.e. *TRO* < 0 or *TRO* > 0). Top row show the empirical distribution of mean FR (gray bars), the fitted single Gaussian probability distribution function (dashed purple line) and the Gaussian mixture distribution function (solid orange line). Middle row show the distribution of Δ*BIC* values under the null-hypothesis (blue bars) and the value of the Δ*BIC* corresponding to the real data set (red square). Bottom row show the average firing rate over time in the time window starting 100 ms before the encoding time window and ending 100 ms after it. The trials were sorted according to the component of the mixture they belong to (i.e. the one for which the posterior probability of the latent variables of the expectation maximization algorithm was higher). Shaded area represent SEM.
(PDF)
