# Supplementary material for: The Encoding of Decision Difficulty and Movement Time in the Primate Premotor Cortex
Source: PLoS Comput Biol. 2015 Nov 10;11(11):e1004502. doi: 10.1371/journal.pcbi.1004502 (PMC4640568; doi:10.1371/journal.pcbi.1004502)
Supplement: S1 Text — We tested the functioning of the Benjamini-Hochberg procedure to control the FDR, varying Q, with synthetic datasets, where the ground truth is known. When no false null-hypotheses is present, no discovery is made over the whole range of Q. When a given number of false null-hypotheses is present, the number of true discoveries is never higher than the number of false null-hypotheses over the whole range of Q. (PDF) [file pcbi.1004502.s001.pdf]

Supplementary Information: False Discovery Rate under Controlled Conditions

The encoding of decision difficulty and movement time in the primate premotor cortex

Marina Martinez-Garcia[1,◑], Andrea Insabato[1,◑,*], Mario Pannunzi[1,◑], Jose L. Pardo-Vazquez[2,3], Carlos Acuña[3], Gustavo Deco[1,4]

1 Universitat Pompeu Fabra, Theoretical and Computational Neuroscience Center for Brain and Cognition, 08018 Barcelona, Spain
2 Circuit Dynamics & Computation Laboratory, Champalimaud Neuroscience Programme, 038 Lisboa, Portugal
3 Departamento de Fisiología, Facultad de Medicina, Universidad de Santiago de Compostela, 15782 Santiago de Compostela, Spain
4 Institució Catalana de Recerca i Estudis Avançats (ICREA), 08010 Barcelona, Spain

◑These authors contributed equally to this work.
*** andrea.insabato@upf.edu**

In this supplementary text connected to the article "The encoding of decision difficulty and movement time in the primate premotor cortex" we want to explore the functioning of Benjamini-Hochberg (BH) procedure to control the False Discovery Rate (FDR) in multiple hypothesis testing under controlled conditions, where we know the ground truth. Here we want to give some insight on the potentiality of this method when the maximum number of accepted false discoveries Q is not fixed. Benjamini-Hochberg procedure can be summarized as following:

- given a list of null-hypotheses $H0_1, \dots, H0_m$ and associated p-values $p_1, \dots, p_m$, order the lists in ascending order of p-values,
- find the largest p-value p_k such that $p_k \leq Qkm^{-1}$, where Q is the maximum FDR we are willing to accept,
- reject all null-hypotheses $H0_1, \dots, H0_k$ and accept all the others.

Choosing the value of Q *a priori* may not be easy, depending on the nature of data, the purpose of the analysis and maybe other factors. For example, in an exploratory analysis, we may be incline to report a large number of positive results (or discoveries) accepting a higher rate of false discoveries instead of reporting no or very few discoveries with a very low rate of false discoveries. The case of our analysis is a more concrete example of this situation. In this work we were looking for neurons encoding the difficulty of the decision in monkeys premotor cortex. However we were not interested in finding the exact number of neurons encoding the difficulty (or even a number higher than a given value). Hence if the analysis, after correcting for multiple testing with $Q = 0.1$, turns out to find, for example, 100 difficulty encoding neurons, 10% of which corresponds to false discoveries, we can still be sure that at least 90 neurons are encoding the difficulty. If, setting $Q = 0.01$, we make no discovery, then the former result with $Q = 0.1$ may be more valuable for future research. If, setting $Q = 0.05$, we make 20 discoveries, we can be sure that 19 of them are true: is it better

to make 19 true discoveries and 1 false discovery or to make 90 true discoveries and 10 false discoveries? Or more in general, what is the maximum number of false discoveries we should accept and what is the minimum number of true discoveries we should find? The answer to this question depends on the purpose of the study and could be given by defining a cost of false discoveries to minimize and a benefit of true discoveries to maximize.

In summary fixing the value of Q beforehand is not straightforward and may restrict the results of the research. Therefore we propose to explore the range of values for Q. This method avoid choosing one single value for Q, supplying information about how the value of Q affects the results. Moreover one value of Q may be chosen afterwards, according to the purposes of the specific research, if further analysis on the set of rejected null-hypotheses is needed.

However we must be sure that the BH procedure is well suited to this approach. For this reason we present here a small study of this method with a synthetic dataset, where we are able to control the ground truth. Here we want to test the functioning of the BH procedure under two conditions:

1. when all the null-hypotheses tested are true,

2. when only a fixed proportion of null-hypotheses are false.

Under condition 1 we want that, for all the possible values of Q, the number of true discoveries, i.e. the difference between the number of discoveries and the number of false discoveries, lies within the error of the number of discoveries, or ideally that none of the null-hypotheses is rejected. Under condition 2 we want that the number of true discoveries is never higher than the number of false null-hypotheses.

To this aim we built a synthetic dataset composed of a one-dimensional dependent variable and one explanatory variable and applied a simple linear regression. This condition mimics the one we have in the analysis presented in the main text but the results are robust to other datasets and statistical tests, as explained below. We created M independent explanatory variables $x_1, \ldots, x_m$, each of them being a random sampling of size N from a uniform distribution between 0 and 1. The corresponding M dependent variables are $y_m = s_m x_m + \eta$ for $m = 1, \ldots, M$, where s_m controls the association between x_m and y_m and η is Gaussian random noise from the distribution $\mathcal{N}(0, 1)$. We then applied a linear model to this dataset. The linear model was $\hat{y_m} = a_0 + a_1 x_m + \xi$, where a_0, a_1 are the coefficients to be estimated and ξ is the residual. After estimating the coefficients of the model, for each variable, we did a t-test in order to test the null-hypothesis that the value of a_1 comes from a normal distribution with mean 0 and unknown variance.

Condition 1 was simulated by setting all $s_m = 0$. In this case there is no association between each pair of variables x_m and y_m, hence all null-hypotheses tested are true by construction. As stated above we want that, for all the possible values of Q, the number of true discoveries lies within the error of the number of discoveries. In fig. 1 we show the number of total discoveries in a bar graph as a function of Q; the red dashed line represents the maximum number of false discoveries, while the black line represents the minimum number of true discoveries, i.e. the difference between total discoveries and the maximum number of false discoveries. We varied the value of N and M in order to show the consistency of the results. It is easily observed that the red line lies always, for all values of Q and all combinations of N and M, within the errorbars of the total number of discoveries, i.e. all null-hypotheses are accepted.

Condition 2 was simulated by setting $s_m = 1$ for $m = 1, \ldots, \lceil 0.2M \rceil$ and $s_m = 0$ for $m = \lceil 0.2M \rceil + 1, \ldots, M$, i.e. 20% of the null-hypotheses is false and the remaining 80% is true. As stated above, under this condition, the number of true discoveries has to be always lower than or equal to the number of false null-hypotheses. Similar to the

previous condition, the results of FDR control for varying value of Q is shown in fig. 2 for different combinations of N and M. Increasing M has the main effect of reducing the fluctuations of results over tests, while increasing N reduces the noise within data of each test and lowers the p-values. The most evident and important observation is that the number of true discoveries (black solid line) is always, for all values of Q and all combinations of N and M, lower than the number of false null-hypotheses in the dataset (blue dashed line). This means that in this case, where we know the ground truth, we can show that even for large values of Q, where we obtain a substantial number of false discoveries, BH procedure successfully controls for FDR and the expected number of true discoveries is never higher than the number of false null-hypotheses. It is also interesting to note that, when the noise in the data is such that p-values are not very small (i.e. small N), small values of Q supply only very few true discoveries compared to number of false null-hypotheses. Therefore a larger value of Q may be more convenient if we wish to maximize the number of true discoveries.

Here we have shown that exploring the range of values for Q may be a meaningful alternative to choosing one value *a priori*.

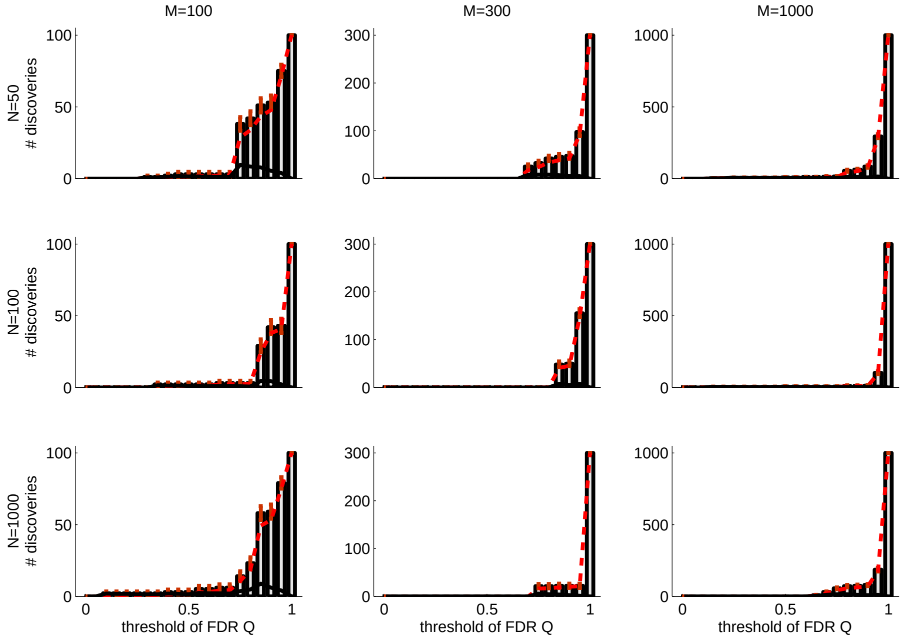

Figure 1. Number of discoveries after controlling for FDR with BH procedure. Each panel depicts a combination of N and M values, as indicated in the figure. In each panel the height of grey bars represents the number of total discoveries (error bars indicate the standard deviation estimated with a binomial distribution). The red dashed line represents the number of false discoveries. The black solid line represents the number of true discoveries, i.e. the difference between total discoveries and false discoveries.

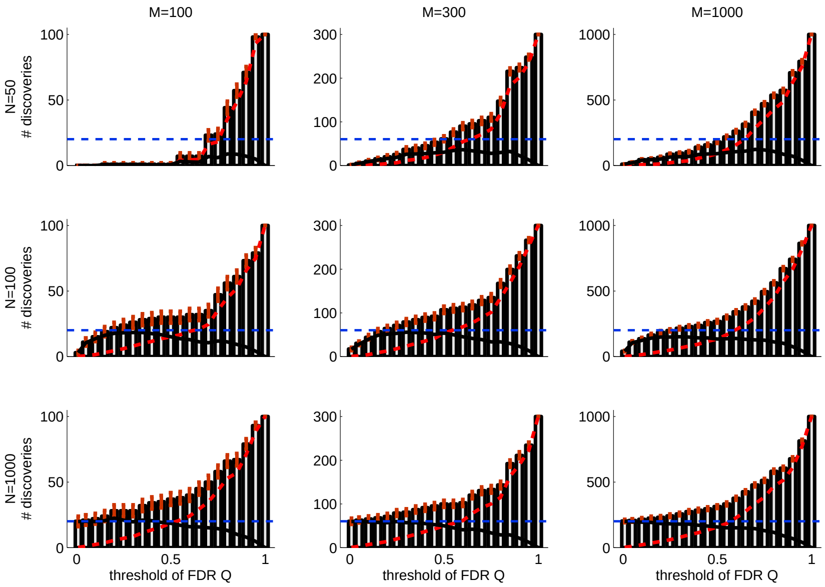

Figure 2. Number of discoveries after controlling for FDR with BH procedure. Each panel depicts a combination of N and M values, as indicated in the figure. In each panel the height of grey bars represents the number of total discoveries (error bars indicate the standard deviation estimated with a binomial distribution). The red dashed line represents the number of false discoveries. The black solid line represents the number of true discoveries, i.e. the difference between total discoveries and false discoveries. The blue dashed line represents the number of false null-hypotheses introduced in the synthetic dataset.
