# Supplementary figures and images for: The Encoding of Decision Difficulty and Movement Time in the Primate Premotor Cortex
Source: PLoS Comput Biol. 2015 Nov 10;11(11):e1004502. doi: 10.1371/journal.pcbi.1004502 (PMC4640568; doi:10.1371/journal.pcbi.1004502)

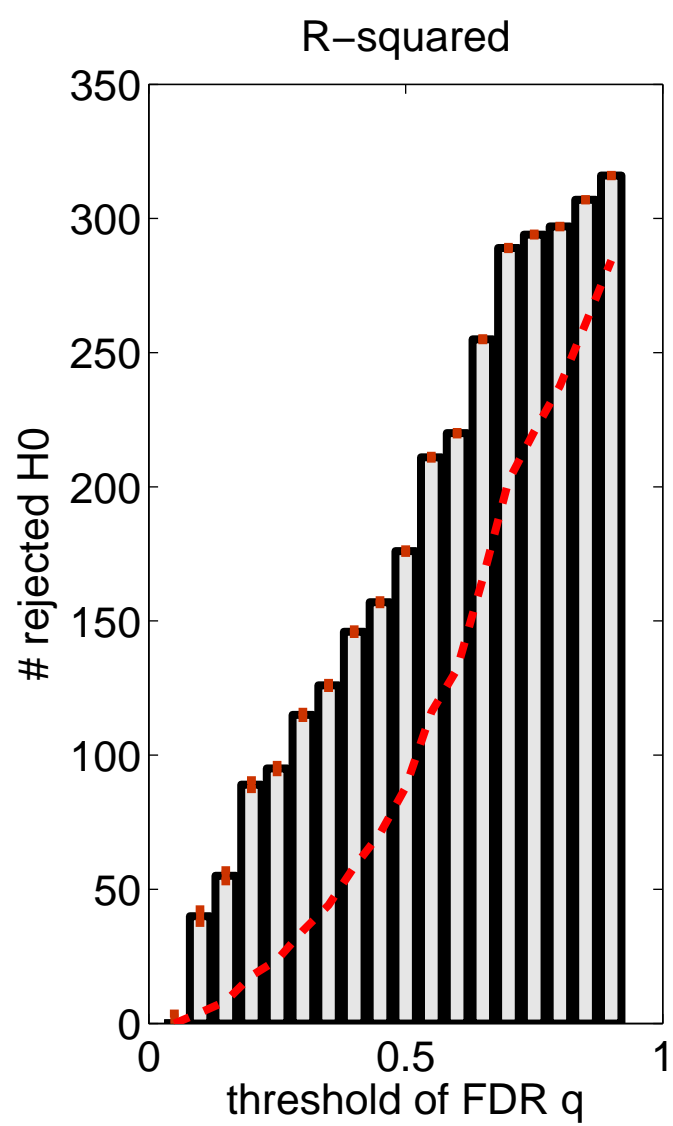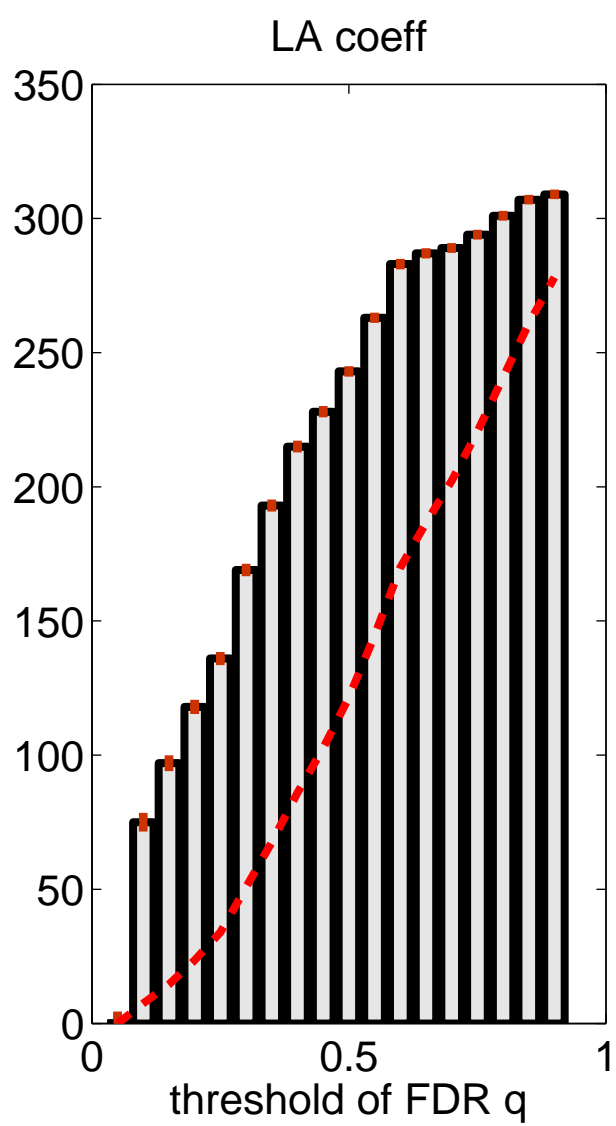

Supplement: S1 Fig — Number of total null hypothesis rejected as a function of Q value for the linear model LM mt. The number of total discoveries is represented by bars (errorbars represent standard deviation of bootstrapped data) as a function of the value of Q. The dashed red line represents the maximum number of accepted false discoveries. (PDF) [file pcbi.1004502.s002.pdf]

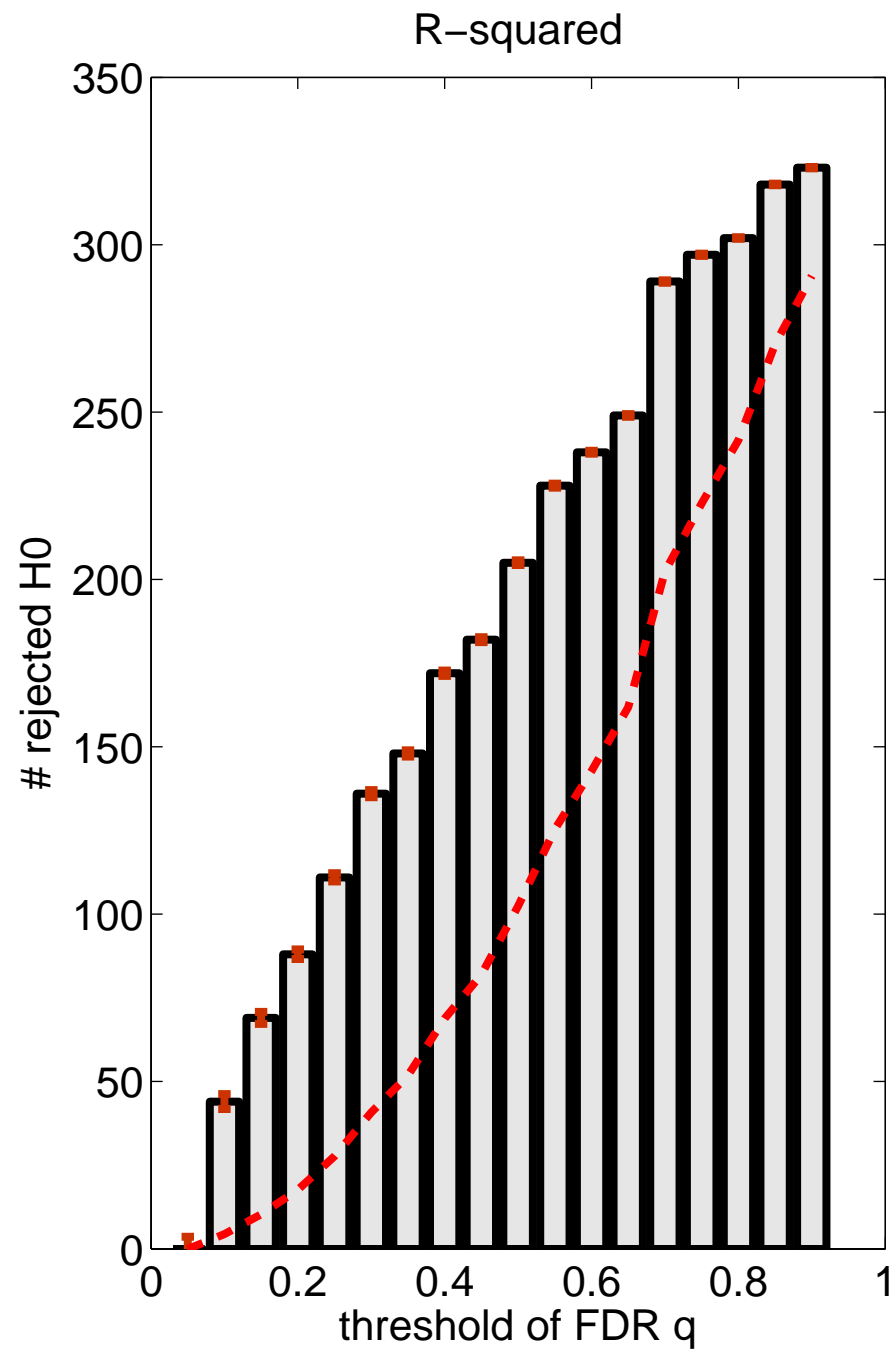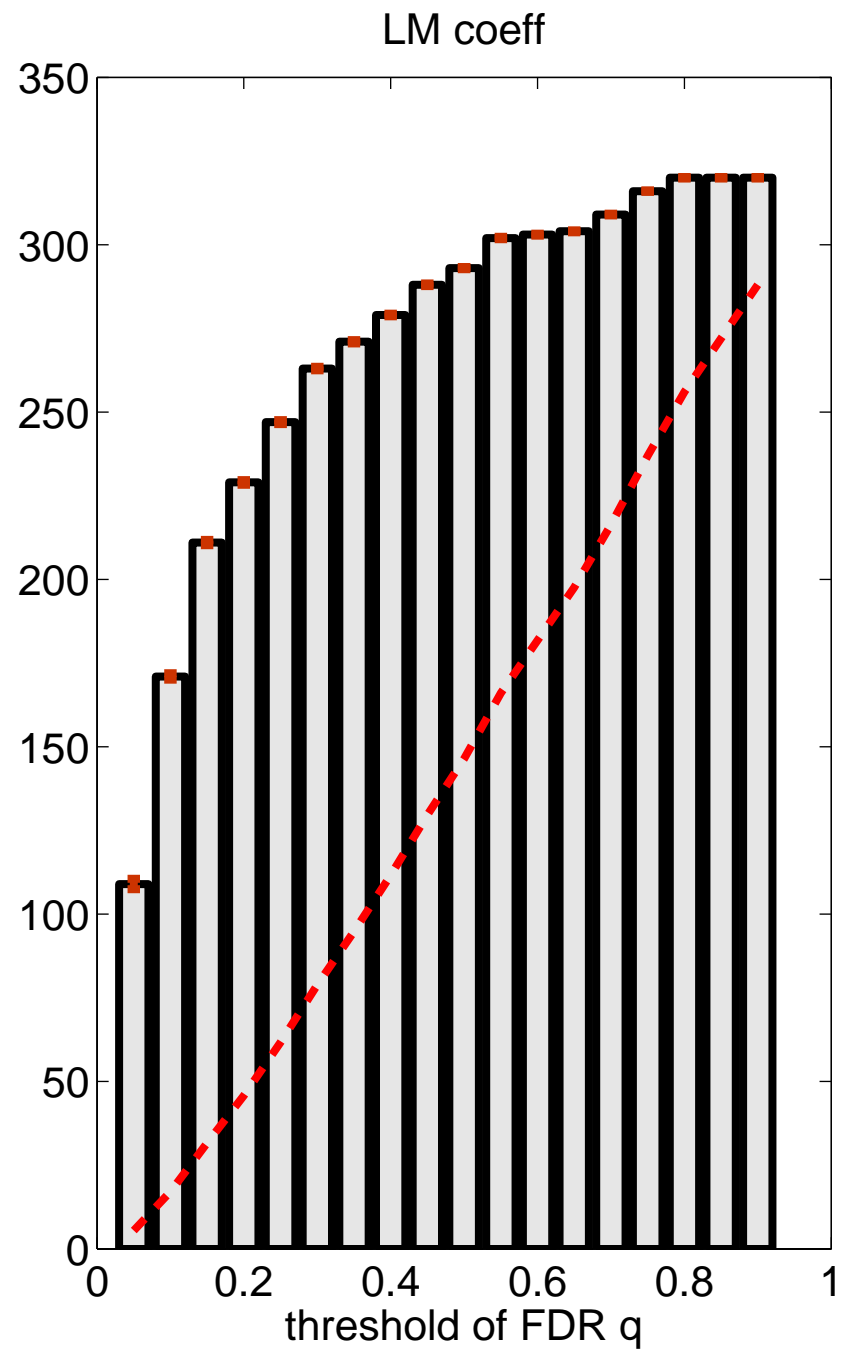

Supplement: S2 Fig — Number of total null hypothesis rejected as a function of Q value for the linear model LM tro and for positive values of TRO. The number of total discoveries is represented by bars (errorbars represent standard deviation of bootstrapped data) as a function of the value of Q. The dashed red line represents the maximum number of accepted false discoveries. (PDF) [file pcbi.1004502.s003.pdf]

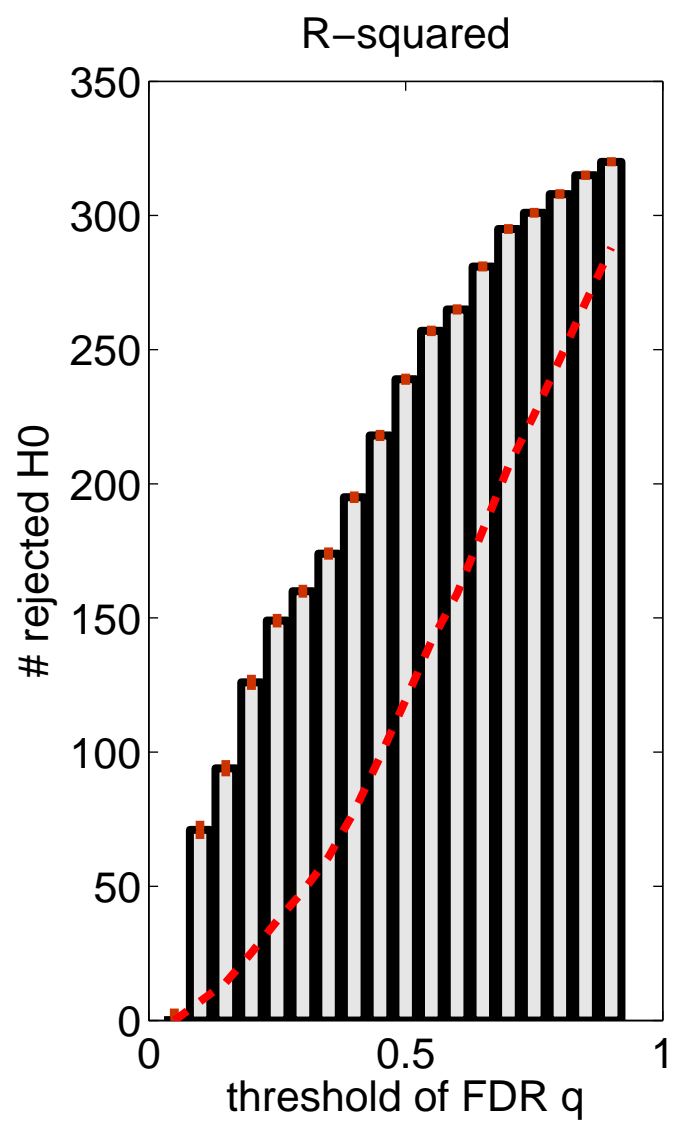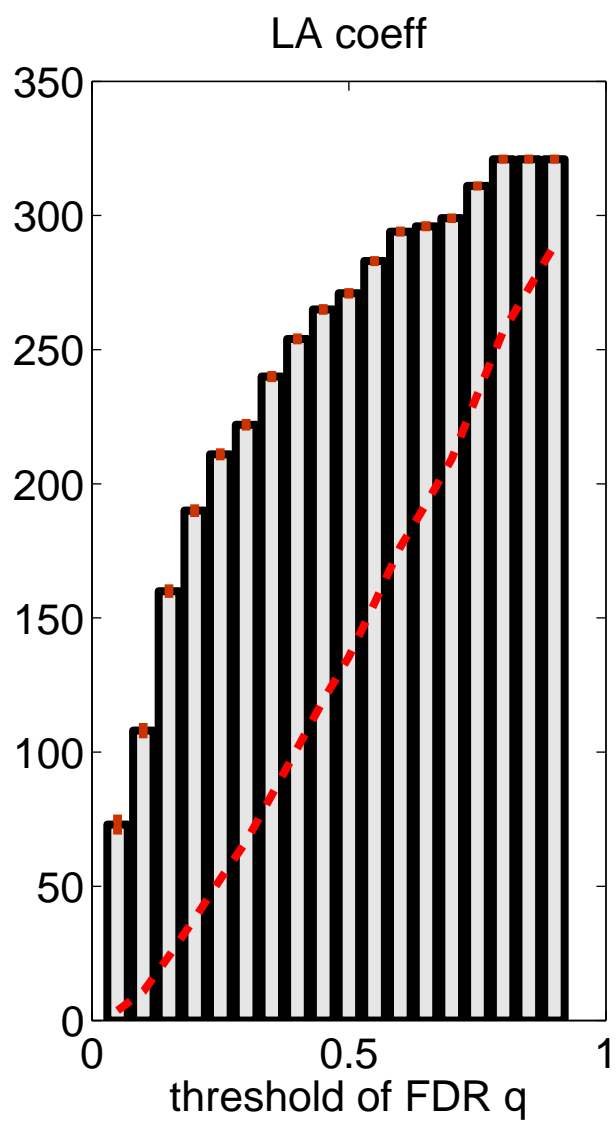

Supplement: S3 Fig — Number of total null hypothesis rejected as a function of Q value for the linear model LM tro and for negative values of TRO. The number of total discoveries is represented by bars (errorbars represent standard deviation of bootstrapped data) as a function of the value of Q. The dashed red line represents the maximum number of accepted false discoveries. (PDF) [file pcbi.1004502.s004.pdf]

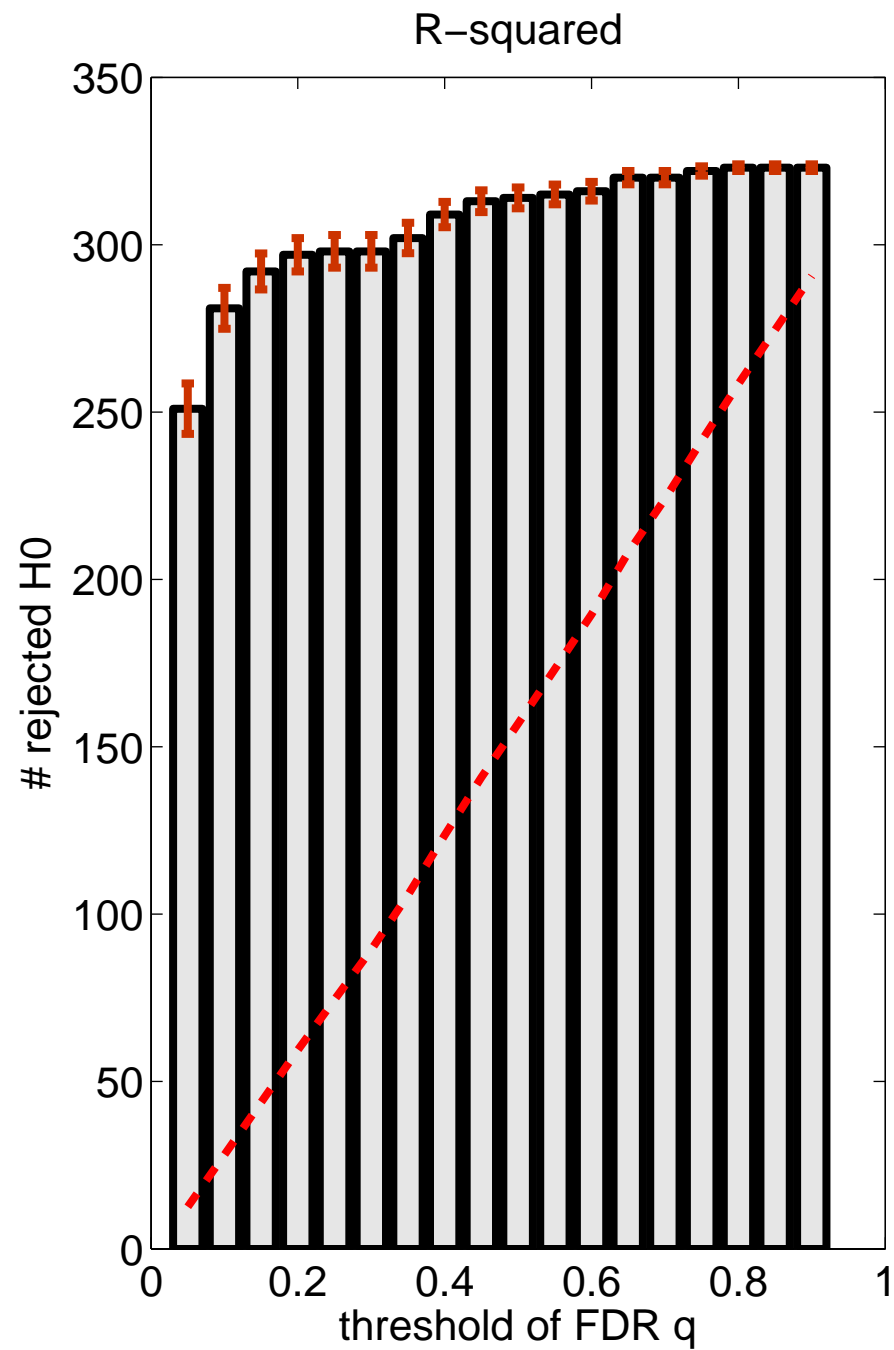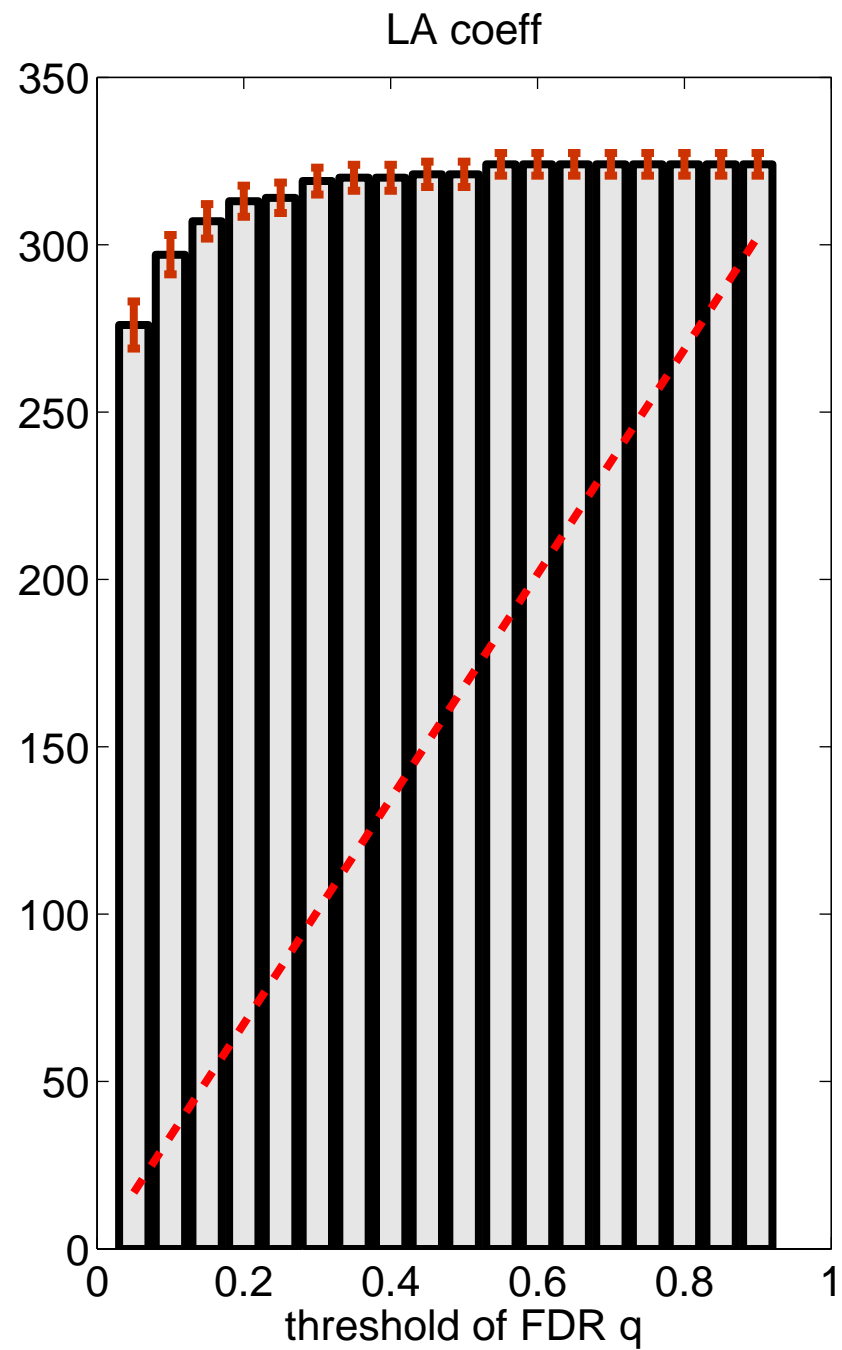

Supplement: S4 Fig — Number of total null hypothesis rejected as a function of Q value for the linear model LM diff. The number of total discoveries is represented by bars (errorbars represent standard deviation of bootstrapped data) as a function of the value of Q. The dashed red line represents the maximum number of accepted false discoveries. (PDF) [file pcbi.1004502.s005.pdf]

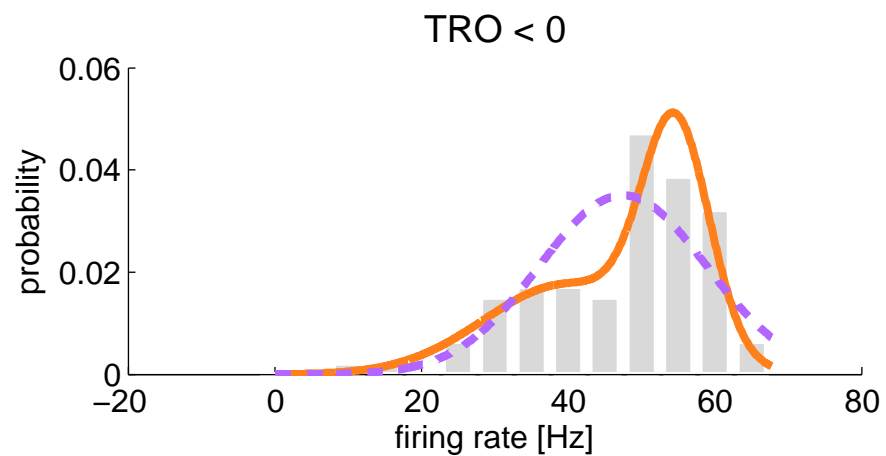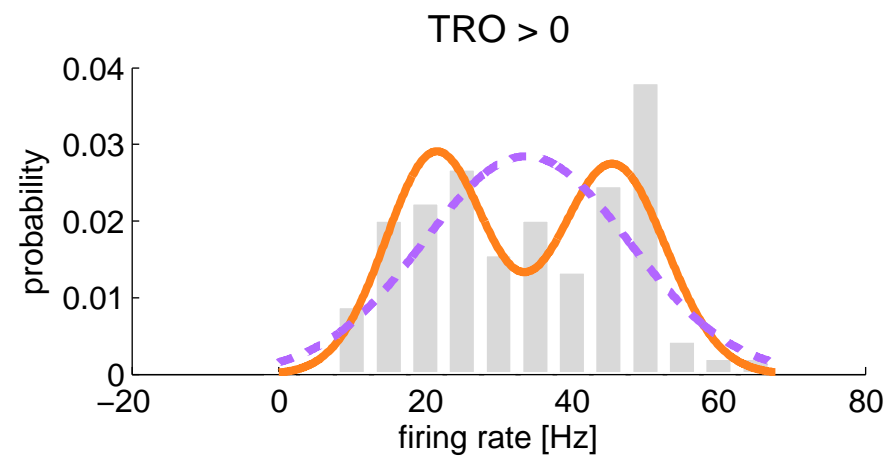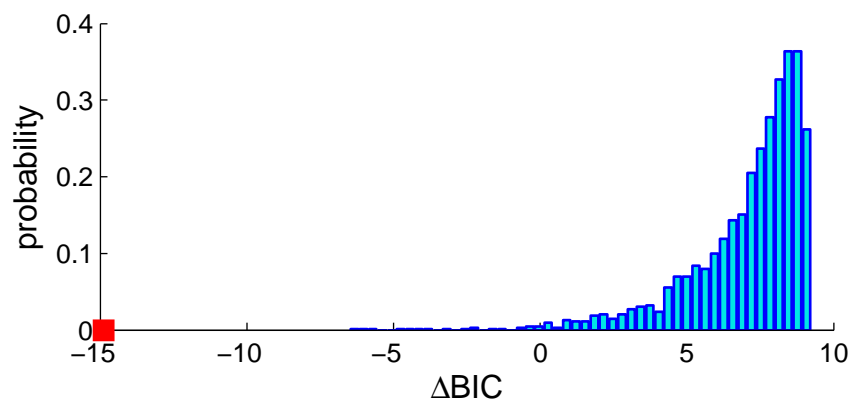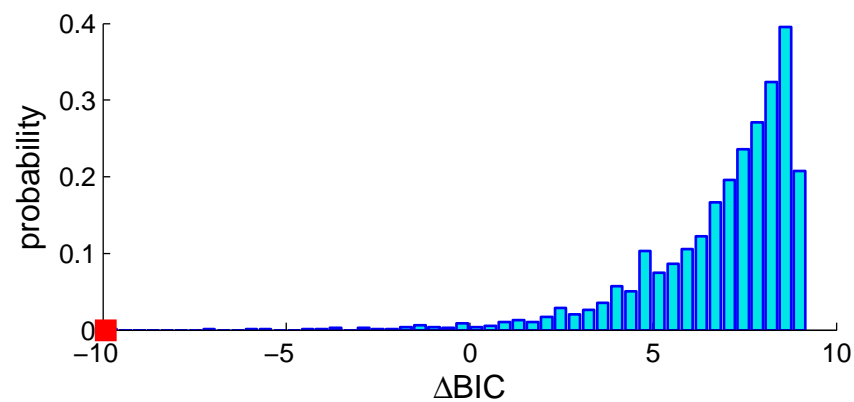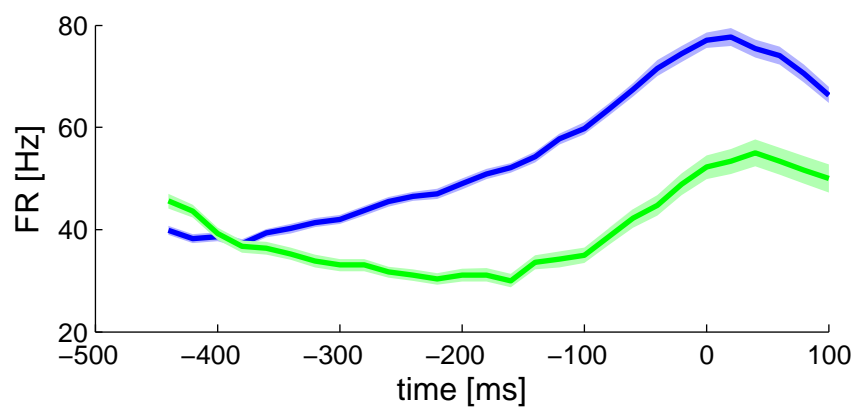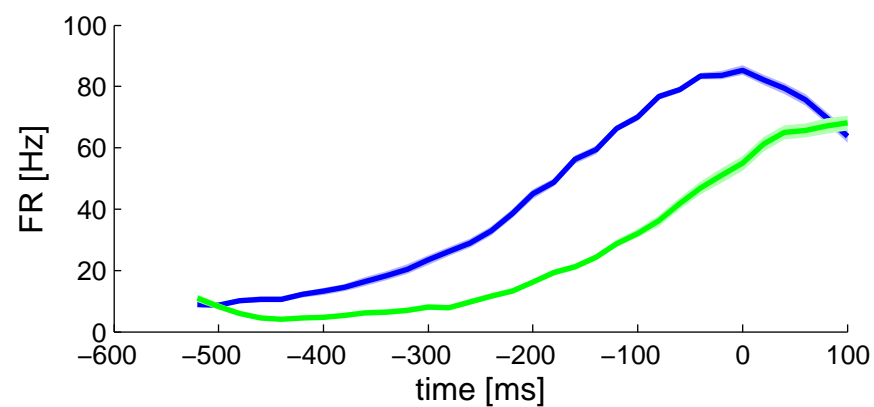

Supplement: S9 Fig — Each column corresponds to one behavioral response (i.e. TRO < 0 or TRO > 0). Top row show the empirical distribution of mean FR (gray bars), the fitted single Gaussian probability distribution function (dashed purple line) and the Gaussian mixture distribution function (solid orange line). Middle row show the distribution of ΔBIC values under the null-hypothesis (blue bars) and the value of the ΔBIC corresponding to the real data set (red square). Bottom row show the average firing rate over time in the time window starting 100 ms before the encoding time window and ending 100 ms after it. The trials were sorted according to the component of the mixture they belong to (i.e. the one for which the posterior probability of the latent variables of the expectation maximization algorithm was higher). Shaded area represent SEM. (PDF) [file pcbi.1004502.s010.pdf]

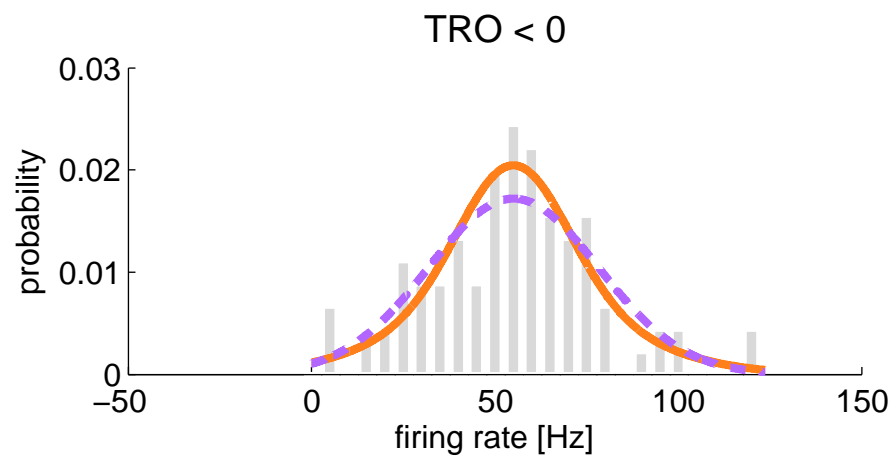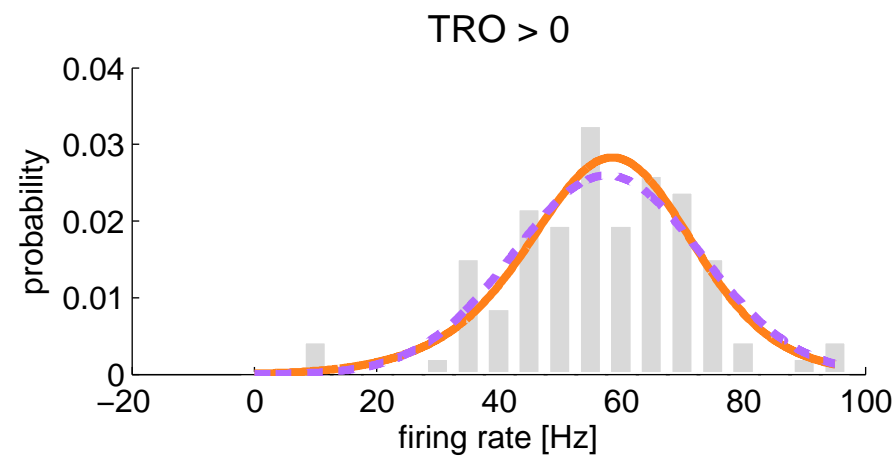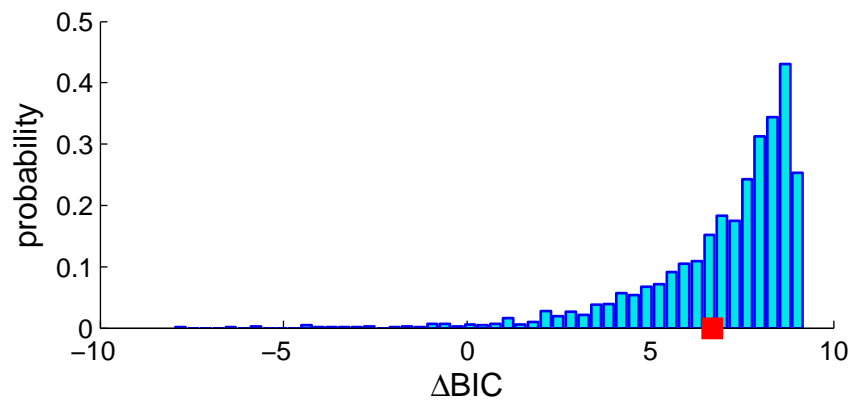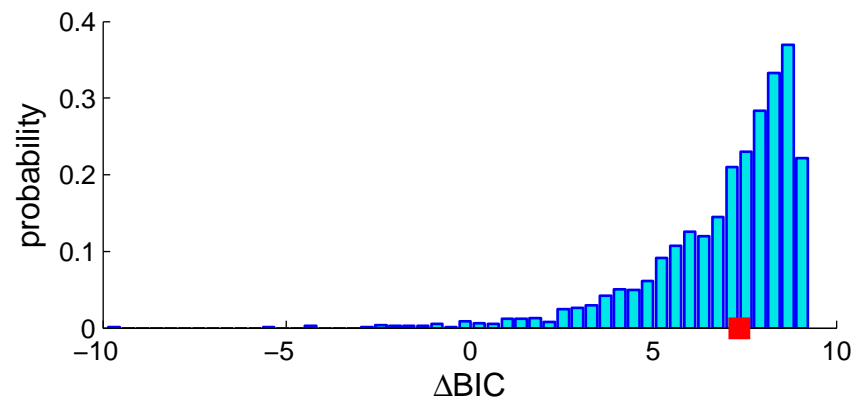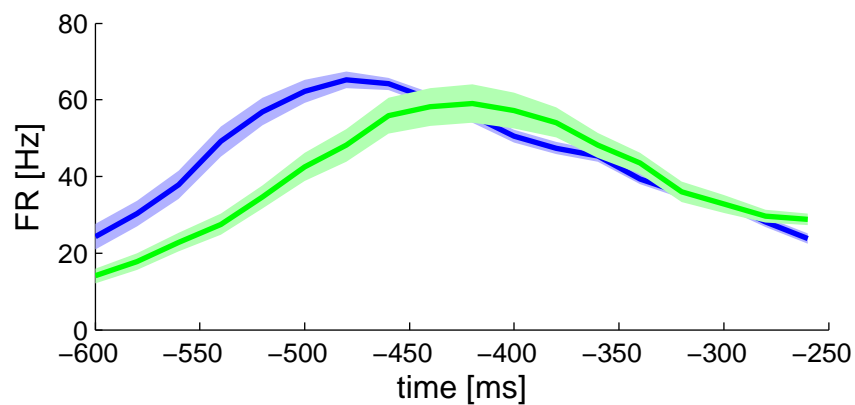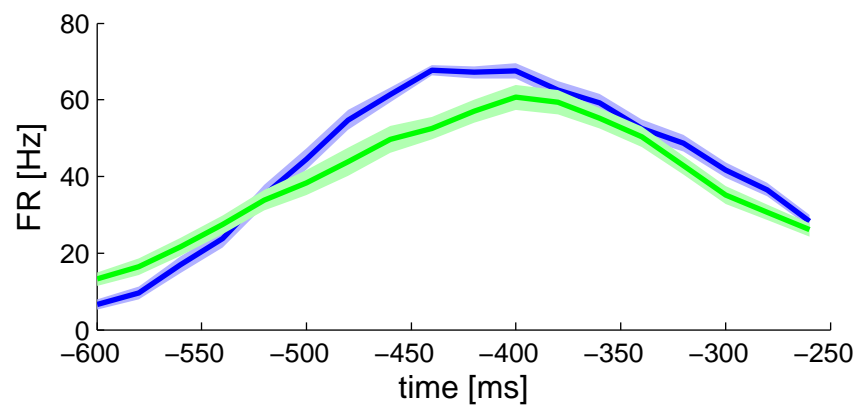

Supplement: S10 Fig — Each column corresponds to one behavioral response (i.e. TRO < 0 or TRO > 0). Top row show the empirical distribution of mean FR (gray bars), the fitted single Gaussian probability distribution function (dashed purple line) and the Gaussian mixture distribution function (solid orange line). Middle row show the distribution of ΔBIC values under the null-hypothesis (blue bars) and the value of the ΔBIC corresponding to the real data set (red square). Bottom row show the average firing rate over time in the time window starting 100 ms before the encoding time window and ending 100 ms after it. The trials were sorted according to the component of the mixture they belong to (i.e. the one for which the posterior probability of the latent variables of the expectation maximization algorithm was higher). Shaded area represent SEM. (PDF) [file pcbi.1004502.s011.pdf]
